# Measuring Spectrotemporal Sensitivity in Cochlear Implant Users With a Reaction-Time Paradigm: A Comparison of Two Implementations

**DOI:** 10.1177/23312165261436198

**Published:** 2026-03-30

**Authors:** Elisabeth Noordanus, Lucas H.M. Mens, Josef Chalupper, Tobias Balkenhol, Marc M. Van Wanrooij, Adrianus John Van Opstal

**Affiliations:** 1 Section Neurophysics, Donders Institute for Brain, Cognition and Behaviour, 198328 Radboud University, Nijmegen, The Netherlands; 2 Department of Otorhinolaryngology, 6034 Radboud University Medical Center, Nijmegen, The Netherlands; 3 Advanced Bionics, 585363 European Research Center, Hannover, Germany; 4 Department of Otorhinolaryngology, 592517 Heilig-Geist Hospital Bensheim, Bensheim, Germany

**Keywords:** spectrotemporal modulation, reaction time, cochlear implant, speech in noise, LATER model, App-based assessment, objective outcome measure, suprathreshold testing, modulation transfer function, auditory psychophysics

## Abstract

This study evaluated two implementations of a reaction-time paradigm to assess spectrotemporal modulation sensitivity in cochlear implant (CI) users, aiming to support both clinical and research applications. Reaction times directly reflect task difficulty, enabling rapid testing with stimuli presented well above modulation detection thresholds. Twenty unilateral CI users completed a task involving the unpredictable onset of broadband and narrowband spectrotemporal modulations embedded in noise. Testing was conducted using two implementations: an app on a smartphone with direct wireless streaming to the CI processor and touchscreen responses (“App”), and a free-field setup with laptop and spacebar responses (“Laptop”), administered 2 to 3 months apart. Speech-in-noise perception was assessed with a matrix test. Reaction times showed strong within-participant consistency across implementations, demonstrating robustness over time and across different delivery and response setups. Individual differences in sensitivity to spectral and temporal modulations were evident and showed strong correspondence between the two implementations. Reaction-time-based modulation transfer functions matched those reported in previous psychophysical studies. Notably, reaction times correlated most strongly (*r* = 0.6–0.7) with speech-in-noise scores for spectrotemporal modulations relevant to speech, particularly spectral densities of 0.25–0.5 cycles/octave combined with temporal rates up to 16 Hz. These findings support the use of reaction times to measure spectrotemporal sensitivity in CI users.

## Introduction

Speech performance in quiet and in noise are important outcome measures of interventions to restore hearing impairment, such as hearing aids and cochlear implants (CIs). To optimize speech perception, it is important to find the optimal individual settings of the hearing device(s). However, speech perception is a complex cognitive skill that not only depends on the integrity of the auditory system, but also on language skills, short-term memory, and other cognitive characteristics of the listener (Blamey et al., 2013; Goupell, 2015; Pisoni et al., 2017). In addition, the effects of interventions may take several weeks to months before speech understanding reaches plateau because CI users need exposure to an extensive set of meaningful speech to fully adapt to a change in device settings (Archer-Boyd et al., 2018). Therefore, speech-in-noise perception may not be an optimal method to evaluate the acute effect of interventions.

Speech consists of joint complex acoustic modulations as a function of time and frequency. Broadband spectrotemporal ripples carry crucial phonological information, including formant and pitch details in the spectral domain, syllable boundaries in the time domain, and formant transitions across the spectrotemporal domain (Elliott & Theunissen, 2009). Assessing sensitivity to spectrotemporal ripples can provide an important measure of the early stages of speech processing, but, ideally, its assessment should rely minimally on the listener's cognitive abilities or language skills (e.g., Kirby et al., 2019). Since spectrotemporal ripples are meaningless sounds for which the listener has little or no reference, higher cognitive effects are expected to be minor (Chi et al., 1999; Van der Willigen et al., 2024). Chi et al. (1999) showed that the detection thresholds for particular ripples relate to the listener's speech-intelligibility performance in noise. Drullman et al. (1994a, 1994b) showed that temporal modulations in filtered voiced signals between 4 and 32 Hz are important for sentence intelligibility, while Elliott and Theunissen (2009) demonstrated that temporal modulations between 1 and 7 Hz, and spectral modulations up to 1 cycle/kHz contributed most to speech comprehension in quiet and in noise. Ripple sensitivity further captures the influence of noise, reverberation and nonlinear distortions like phase-jitter and phase-shifts (Elhilali et al., 2003).

Spectrotemporal modulation sensitivity has also been measured to understand auditory processing in hearing-impaired listeners using a hearing aid or a CI, showing a significant relationship with speech understanding (Holden et al., 2016; Lawler et al., 2017; Zaar et al., 2023, 2024; Zheng et al., 2017; Zhou, 2017). Sensitivity to spectral and temporal modulations could therefore be considered as an early predictor for speech understanding after clinical interventions (Henry & Turner, 2003).

Ripples have traditionally been used in alternative forced choice (AFC) discrimination tasks. Reaction times (RTs) to ripples are an alternative option, providing a measure for the sensory-neural processing speed from stimulus presentation to motor response. Although RT is a widely used performance metric in visual-motor psychophysics, it has rarely been used in auditory research, or in audiological clinical practice. Notable exceptions include Hofman and Van Opstal (1998), who investigated the effect of sound-burst duration on RTs in goal-directed eye movements; Schlittenlacher et al. (2017), who examined how stimulus loudness influences detection difficulty; Veugen et al. (2022) and Van der Willigen et al. (2024), who validated RTs to the onset of spectrotemporal ripples as a measure of spectrotemporal sensitivity in both normal-hearing humans and monkeys; and Noordanus et al. (2025), who measured RTs of CI users to changes in electrode or modulation frequency using direct electrode stimulation. Because the reaction-time paradigm measures the detection of a change in the sound, it places minimal demands on working memory. As the task instruction is simple (“press the button as fast as possible when you perceive a change”), procedural learning effects on the results (e.g., De Jong et al., 2018) can be minimized (Van der Willigen et al., 2024). Furthermore, presenting the relevant stimulus features well above the discrimination threshold can help reduce mental fatigue, improve reliability, and more accurately simulate real-world conditions, compared to measurements taken at threshold levels. Reaction-times allow for fast testing, whereby the measurement time can be as short as 1 min to collect 20 responses to reliably determine sensitivity for a certain spectrotemporal modulation (Van der Willigen et al., 2024; Veugen et al., 2022). This makes it feasible to use RTs for spectrotemporal ripples to determine a listener's spectrotemporal sensitivity in clinical practice.

Conceptually, the RT comprises three components that form a serial processing chain: the sensory stage (including cochlea, auditory nerve, and early subcortical nuclei), a central processing stage that performs binaural integration, conscious stimulus detection, identification and decision making at the cortical level, and the motor execution stage resulting in an overt response once the decision to respond has been made (Donders, 1969; Noorani & Carpenter, 2016). The total RT for a manual response to a stimulus generally varies from 200 to 400 ms. Since the combined time required for sensory processing and motor execution is only about 50 ms (auditory system) or 100 ms (visual system), the decision time needed by the central stages in the brain is considerably longer—and also more variable, both within and between participants—than the sensory- and motor conduction times ( Carpenter and Williams, 1995; Noorani & Carpenter, 2016). The LATER model of Noorani and Carpenter (2016) describes how uncertainty and internal neural noise influence reaction-time distributions (see Supplemental Material S1).

In this paper, we aim to investigate the efficacy of reaction-time-based measurements evoked by spectrotemporal modulations, referred to as STM-RT, for assessing the spectrotemporal sensitivity of CI users. Our approach involves three key objectives: (i) evaluating the consistency of individual response patterns across two different implementations, (ii) comparing the results with established modulation transfer functions (MTFs) for spectrotemporal ripples obtained with other psychophysical tests, and (iii) verifying the correlation between reaction-time-measured spectrotemporal modulation sensitivity (a low-level auditory processing measure) and speech-in-noise perception (a high-level auditory processing measure).

To achieve these objectives, we collected manual RTs from CI users and compared two implementations of the STM-RT method. In the “App” implementation, the participant was sitting alone in an office and the spectrotemporal ripple was provided to the user's CI through wireless direct audio from a smartphone. The RT was detected as a hit on the touchscreen. A reliable self-test tool could make it feasible to extensively probe the spectrotemporal modulation sensitivity of CI users. This is important because—compared to normal-hearing listeners—CI users may show large irregularities in hearing, for example, caused by the algorithms of the processor, or due to differences in sensory-neural integrity. Accurate mapping of these irregularities may help to develop individualized signal processing and fitting approaches. A self-test tool further promises to enable frequent monitoring of the spectrotemporal resolution over time in the home situation.

For the “Laptop” implementation, the tests were performed in the presence of the researcher in the lab and employed conventional free-field acoustical stimulation. Here, RTs were measured by a spacebar press on a laptop.

The two implementations were measured 2 to 3 months apart to evaluate the comparability, reproducibility, and stability of the results. We included broadband (Van der Willigen et al., 2024) and narrowband (1-octave) ripples (Mehraei et al., 2014) to also determine spectrotemporal sensitivity for different frequency bands. All tests were performed under monaural listening conditions with any hearing aid or CI in the other ear switched off, and that ear plugged. We analyzed and compared the reaction-time distributions for both implementations. In addition, speech-in-noise perception was measured in the free field, and its relationship with spectrotemporal-modulation sensitivity was evaluated by calculating correlations for both implementations.

## Methods

### Participants

Twenty-one postlingually deaf CI users (5 female) with a HiRes 90K or Ultra (3D) implant of Advanced Bionics participated. Their demographic details are listed in Table 1. All participants were native Dutch speakers. Participant S7 withdrew from the study after the first session. Most users were bimodal listeners. All tests were performed monaurally, with the contralateral device switched off.

**Table 1.** Demographic Details.

Participant	Speech Perception	Age	CI Use [years]	Device Type (electrode)	
				Tested Ear	Non-tested Ear
S1	Medium	60–69	9	90 K (HiFocus Helix)	HA
S2	Medium	70–78	5	90 K Advantage (HiFocus ms)	-
S3	Good	60–69	15	90 K (HiFocus 1J)	HA
S4	Poor	70–78	15	90 K (HiFocus Helix)	HA
S5	Medium	35–39	8	90 K Advantage (HiFocus ms)	HA
S6	Poor	60–69	3	Ultra 3D (HiFocus ms)	HA
S7*	Medium-poor	50–59	4	90 K Advantage (HiFocus ms)	HA
S8**	Good	60–69	2^†^	Ultra 3D (HiFocus SlimJ)	HA
S9	Medium	60–69	6	90 K Advantage (HiFocus ms)	HA
S10	Medium	79–82	3	Ultra (HiFocus ms)	HA
S11	Poor	70–78	4	Ultra (HiFocus ms)	HA
S12	Medium	70–78	0.7	Ultra 3D (HiFocus ms)	HA
S13	Poor	70–78	4	90 K Advantage (HiFocus ms)	HA
S14	Medium-poor	50–59	1	Ultra 3D (HiFocus SlimJ)	HA
S15***	Medium-poor	60–69	16	90 K (HiFocus 1J)	-
S16	Medium	60–69	2	Ultra 3D (HiFocus ms)	HA
S17	Good	35–39	8	90 K Advantage (HiFocus ms)	CROS
S18	Good	50–59	7	90 K Advantage (HiFocus ms)	HA
S19*	Poor	79–82	11	90 K (HiFocus 1J)	C1
S20	Good	50–59	7	90 K Advantage (HiFocus ms)	HA
S21	Poor	79–82	2	Ultra 3D (HiFocus ms)	HA

Speech-perception column: informal categorization within this cohort; 50% speech-in-noise perception thresholds obtained with the speech-in-noise matrix test are shown in Figure 6. Good: speech perception threshold (SNR) < 0 dB SNR; medium: 0–3 dB; medium-poor: 3–5 dB; poor: > 5 dB. CI use: duration of cochlear implant use. HA: hearing aid. The device in the non-tested ear (rightmost column) was switched off, and the ear was plugged. ^†^: Reimplantation 0.5 years ago. *: excluded from comparisons: S7 withdrew after session 1; for S19 no 50% speech-in-noise reception threshold could be established; for this participant electrode 11–16 were deactivated. **: completed one STM-RT block instead of two in session 2. ***: electrodes 15 and 16 were deactivated.

This study fully adhered to the ethical approval NL74369.091.20, issued by the Ethical Committee of Radboudumc (Commissie Mensgebonden Onderzoek Regio Arnhem-Nijmegen). We obtained informed written consent from each participant prior to the measurements.

### Experimental Setup

We used the same Naída CI Q90 Sound Processor in all experiments, fitted for each participant with the device settings as preferred for everyday use. For the free-field Laptop measurements, the CI processor microphone was used in Omni Directional mode. Volume control and pre-processing features (WindBlock, SoundRelax and EchoBlock) were disabled. The device in the other ear (if present) was switched off or the device was removed, and the ear plugged in case of any residual hearing. The participant used the preferred hand to tap on the screen (App) or hit the spacebar (Laptop).

#### App Implementation

In the “App” implementation, a new research app of Advanced Bionics (TRaM—Test, Rate and Monitor) on an iPhone 6s (Apple, Cupertino, CA, USA) prepared the sound and transmitted it via Bluetooth to the Phonak ComPilot, which streamed it to the CI sound processor through 10.6 MHz radio frequency technology. The “Audio mixing” of the sound processor was set to “100% ComPilot.” All App measurements were performed with the same iPhone 6s mobile phone. The sound level was calibrated by reading out the input level meter of the automatic gain control (AGC) of the Naída Sound Processor, a process that only needed to be performed once. The iPhone output level was fixed at the maximum setting (100%). The participant was sitting alone in a standard office room while performing the tests.

#### Laptop Implementation

In the “Laptop” implementation, we presented the sound from a speaker at the straight-ahead location (Tannoy Reveal 402). The Babyface soundcard of RME (Audio AG, Germany) transferred the sound to the speaker. A custom-written MATLAB application (The MathWorks, Natick, MA, USA, version R2018a–R2019b) generated the stimuli and detected the spacebar hit. Measurements were performed in a sound-treated normally lit room, in which the background noise level was less than 25 dBA. The sound level was calibrated using the SLM 1352P sound level meter (Isotech, Southport, England). The researcher was sitting next to the participant, answering any questions in between measurement blocks.

### STM-RT Test

#### Stimuli

Table 2 gives an overview of the stimuli: broadband ripples (250 Hz–8 kHz passband) and four narrowband ripples with a bandwidth of 1-octave geometrically centered at 500 Hz, 1, 2, and 4 kHz, respectively. The stimulus set was restricted to keep each presentation block (10 repetitions per stimulus) within approximately 10 min. Therefore, rather than testing the full spectral × temporal modulation matrix, we selected the combinations expected to be most informative for distinguishing performance within and between participants and for correlating with speech-in-noise outcomes, based on Veugen (2017). All ripples used a modulation depth of 82% to permit presentation of the full intended modulation range, with modulation minima set to levels expected to remain audible after AGC processing. Each stimulus consisted of pink noise, bandpass filtered from 0.1 to 8.7 kHz using a first-order Butterworth filter (Mehraei et al., 2014). After a randomly selected duration between 700 and 1200 ms (in 100 ms steps), sinusoidal modulation within the target bandwidth commenced instantaneously, without ramping, with a starting phase of 0 rad, and applied on a linear amplitude scale. The modulation had a maximum duration of 3 s, and each trial was terminated immediately upon a response. The pink noise served to prevent spurious cues, such as edge effects around ripple onset (Archer-Boyd et al., 2018; Aronoff & Landsberger, 2013; Winn & O’Brien, 2019). The bandwidth of the ripples was accentuated with 15 dB relative to the surrounding noise for the full duration of the stimulus (see below for presentation levels). This accentuation was applied as Mehraei et al. (2014) found that it was needed to achieve sufficient audibility of all ripples for some hearing-impaired listeners. The spectral boundaries of this accentuation were smoothed using a Hanning window with a width of 100 Hz. In the Laptop measurements each trial was generated real-time, with different carrier noise in every trial (fresh noise). The App stimulus set was prepared offline with the same software; for each stimulus, two noise renderings were generated and presented in random order during the experiment.

**Table 2.** Overview of the Ripple Stimuli With Spectral and/or Temporal Modulations.

**Narrowband (1-octave), 4 Hz, 2 cycles / octave (c/o)**				
**Center freq.**	500 Hz	1 kHz	2 kHz	4 kHz
**Broadband**				
	**Temporal modulation frequency** [Hz]			
	**0**	**4**	**8**	**16**
**Spectral modulation frequency** [cycles / octave]	2	2	-	-
	1	1	1	-
	0.5	0.5	0.5	-
	0.25	-	-	0.25
	*catch*	0	0	-

Upper row: Narrowband ripples 1-octave wide centered at resp. 500 Hz, 1 kHz, 2 kHz, 4 kHz; all modulated at [4 Hz, 2 c/o]. Lower part: ripples constructed from broadband pink noise (250 Hz–8 kHz passband); columns show the temporal modulation values in Hz and rows the spectral modulation frequencies in cycles per octave. A dash (–) indicates spectrotemporal combinations not included in the stimulus set; the catch stimulus ([0 Hz, 0 c/o]) is shown in italics. c/o: cycles per octave.

**Figure 1.**
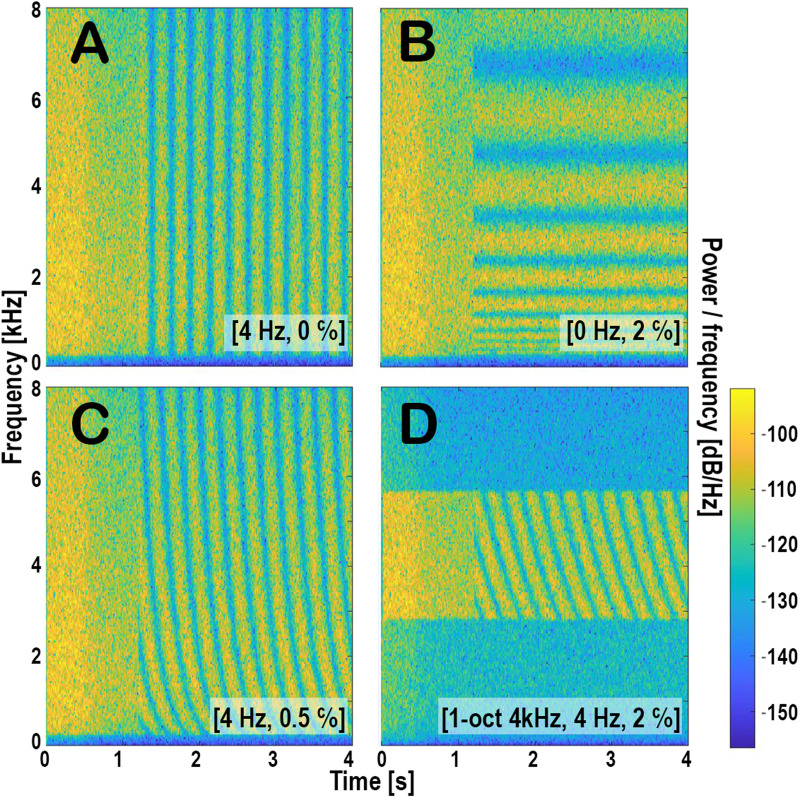
Spectrograms of four examples of the stimuli. (A-C) Broadband ripple modulated at [4 Hz, 0 c/o], [0 Hz, 2 c/o], and [4 Hz, 0.5 c/o], respectively. (D) One-octave wide ripple with a center frequency of 4 kHz, modulated at [4 Hz, 2 c/o]. The 15 dB accentuation of the ripple bandwidth is clearly visible in panel D, present both before and after modulation onset. In all panels, a decrease of intensity caused by the AGC is visible about 400 ms after stimulus onset.

The noise outside the modulated band was presented at 60 dB SPL. Because the modulated band was accentuated by 15 dB—both before and after modulation onset—the overall level of the broadband (BB) stimuli reached 74 dB SPL. The narrowband stimuli had a lower overall level of 68 dB SPL, as the accentuated bandwidth was narrower. In the speech processor, the volume was affected by the AGC. We simulated the effect of AGC (Dwyer et al., 2021) using a MATLAB script provided by Advanced Bionics. The AGC engages approximately 400 ms after the onset of the noise, while the modulation begins no earlier than at 700 ms, ensuring that the AGC has fully stabilized before modulation onset. After stabilization, the output sound level of the broadband stimuli was 56 dB SPL. For the narrowband stimuli, stabilized output levels were 55 dB SPL for the 4 kHz centered ripple and 54 dB SPL for the other narrowband conditions. These small differences arise from frequency-dependent attenuation introduced by the speech processor's pre-emphasis filter. Importantly, because the AGC was already fully engaged prior to modulation onset and remained stable throughout stimulus presentation, no additional loudness changes occurred when the modulation was introduced. Figure 1 shows the spectrograms of four stimuli, including the effect of AGC.

A broadband catch stimulus was included to estimate each participant's false positive response rate and to account for potential unintended acoustic cues arising from technical imperfections—such as activation of the AGC—that could elicit a response. This stimulus was generated identically to the others, except that both temporal and spectral modulations were set to zero ([0 Hz, 0 c/o], Table 2; c/o: cycles/octave).

#### Task

Participants were instructed to react as soon as they perceived a change in the sound. They were further informed that some stimuli might contain no change at all. In the App implementation, they were additionally informed that performance feedback would be shown after each trial: a green smiley for quick responses (within 350 ms), an orange smiley for slower responses, and a red smiley for responses made before modulation onset or during presentation of the catch stimulus. In the Laptop application, no feedback was provided. Each trial was terminated immediately upon a response or after 3 s. In case the participant responded before modulation onset, the trial was reiterated. The inter-trial interval was 0.5 s.

### Speech-in-Noise Test

The speech material was based on the Dutch version of the speech-in-noise matrix test developed by Houben et al. (2014). The test uses sentences composed from a fixed matrix of words. All sentences shared the same grammatical structure (name, verb, numeral, adjective, object), but were semantically unpredictable (Dietz et al., 2014; Van Groesen et al., 2023). The occurrence of phonemes matched that of standard Dutch. Thirteen different lists, each containing 20 unique five-word sentences, were created to allow repeated testing. We used three of these lists, in which each of the 50 words from the matrix occurred twice. A subset of these data, from 16 participants, was also used in Noordanus et al. (2025).

Participants viewed a 10 × 5 word matrix on an A4 sheet and spoke the words aloud; the researcher recorded their responses. Participants were instructed to select one word from each column and were encouraged to guess when uncertain. In case a participant did not select a word from a column, the researcher noted the first word in that column. Therefore, the guessing probability for each word was 1/10.

Sound was presented via a front-facing speaker, as in the Laptop STM-RT implementation. Speech-shaped stationary noise remained fixed at 60 dB SPL, while the speech level was adapted, targeting at the 50% threshold of speech intelligibility in noise. The first sentence was presented with a signal-to-noise ratio (SNR) of +10 dB and the level was reduced when at least three words were correctly selected by the participant. A maximum likelihood adaptive procedure was used, and a psychometric function was fitted after 20 sentences. If the fit error was large, the 50% threshold was estimated by averaging the final 8 presentation SNRs. Further details are provided in Supplemental Material S2.

The results of participant S19 could not be used, as the score remained below 50% even at the maximum speaker output. Consequently, no 50% speech intelligibility level could be determined for this participant. The speech test was not conducted for S7, who withdrew after the first session (see Participants).

### Measurement Order

The STM-RT measurements were performed in two sessions. Session 2 was performed 2 to 3 months after session 1. In session 1, the App implementation was used, and in session 2, the Laptop implementation. Each session included two STM-RT blocks. The time between the two blocks was between half an hour and an hour. At the start of the App experiments, a couple of easily recognizable purely temporal, spectral and spectrotemporal stimuli (e.g., [8 Hz, 0 c/o], [0 Hz, 0.5 c/o], [4 Hz, 0.5 c/o]) was played as a demonstration, followed by a training block to familiarize the participant with the STM-RT paradigm, and using the iPhone speaker to allow communication between the participant and the researcher.

Each STM-RT block consisted of 10 presentations (trials) of the 17 ripple stimuli (Table 2) in a pseudorandom order. The testing time for the 170 trials was between 9 and 11 min, around 35 s per stimulus (10 trials), depending on how many of the stimuli could be detected by the participant, because the trial was immediately aborted upon a response. In the App implementation, all 170 trials of one block were presented without a break. In the Laptop implementation, the presentation was halted after every 20 trials until the participant hit the spacebar to continue; most participants immediately continued after 20 trials without pausing. The laptop screen showed a progress bar from 1 to 170; several participants reported that they found this helpful.

The speech-in-noise measurements were performed in session 2. The training consisted of one speech-in-noise list, the test of two lists. All participants had previous experience with the matrix test. The average duration of the speech-in-noise test was 5 min.

All participants completed session 1; S7 did not perform session 2, and for S8 only one STM-RT block was measured in session 2 due to time constraints. For S8, the fitting of the M-levels had been adjusted by the clinician between session 1 and 2—showing a considerable overall increase, with only the basal electrodes left unchanged.

### Processing STM-RT Measurements

RT was defined as the interval from the onset of the modulation (in the case of the catch stimulus the [0 Hz, 0 c/o] “modulation” described above) to the participant's response. RTs exceeding 2500 ms after modulation onset (for the App, after delay correction; see below), as well as non-responses were coded as 2500 ms. Responses faster than 140 ms were removed—except indicated otherwise—as these are generally considered predictive or accidental instead of a true reaction to the onset of the modulation (Green & von Gierke, 1984; Thompson et al., 1992). We pooled the data for each stimulus from the two blocks and calculated the median RT.

**Figure 2.**
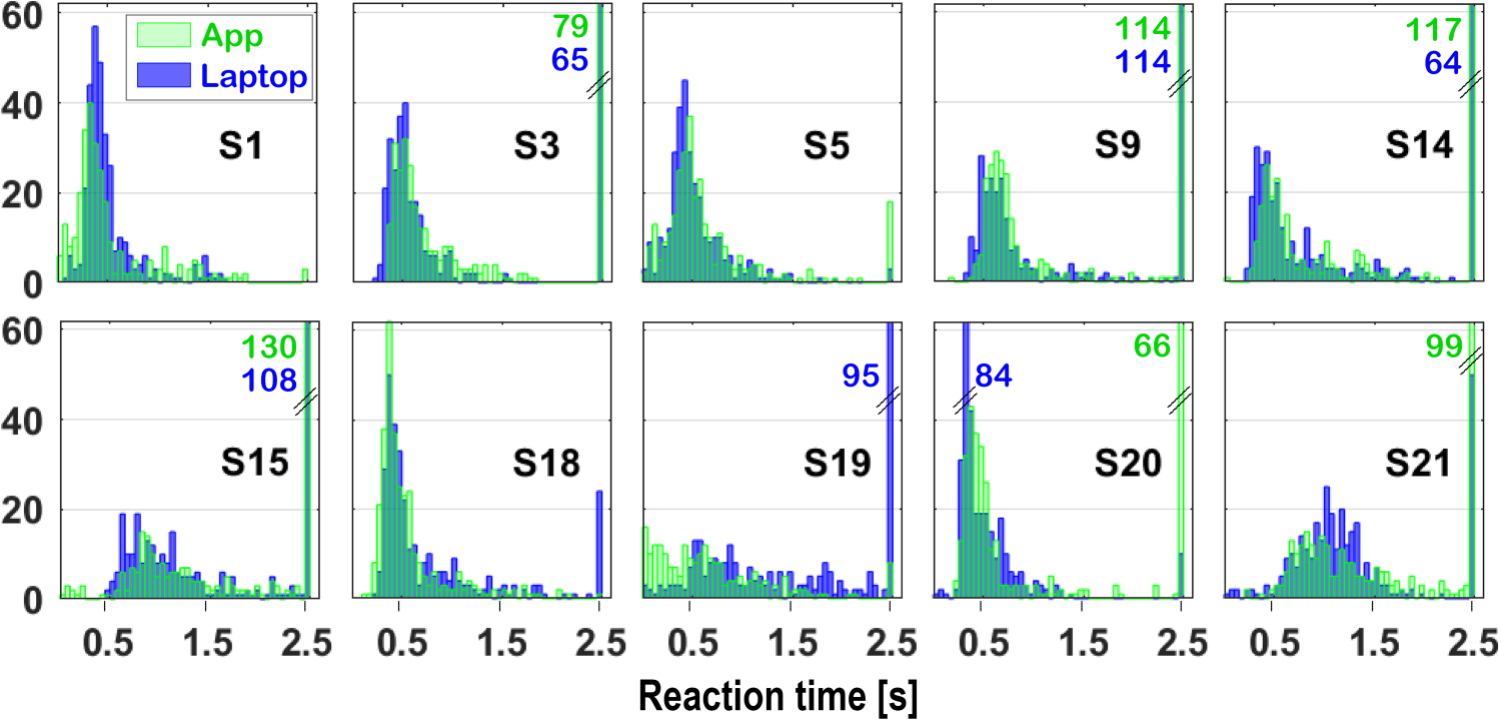
Raw histograms for 10 participants of all reaction times (0 < RT ≤ 2.5 s, responses > 2.5 s and non-responses were set to 2.5 s); responses to all stimuli (excluding the catch stimulus) were pooled. Bin counts exceeding 62 are indicated in the plots. App data are shown in transparent green, Laptop data in blue. Note that for most cases, distributions look qualitatively similar for the two measurement implementations. Participant S19 did not yet fully understand the task during the App measurements and responded to the onset of the noise rather than to the modulation onset, resulting in many very short reaction times. In contrast, the Laptop measurements in session 2 showed many non-responses, consistent with correct task execution for this participant (a poor speech performer, see Table 1).

Results obtained with the Laptop and App implementations were compared, excluding participants S7 and S19. Participant S7 did not complete the Laptop measurements (see above), and participant S19 did not correctly understand the STM-RT task during the first session (see Figure 2). The Results section therefore includes data from the remaining 19 participants, unless stated otherwise.

#### Reciprobit Plots

We used reciprobit plots to graphically represent the distribution of RTs for a particular stimulus and participant (Supplemental material, Figure S1B, C). In these plots, cumulative response probability is displayed on a probit scale and plotted against promptness, defined as the reciprocal of the reaction time (1/RT). Under the LATER model (Noorani & Carpenter, 2016; Supplemental material S1), a Gaussian distribution of internal evidence rates appears as a linear relationship in this representation.

The promptness axis was flipped, so that faster responses are located on the left and slower responses on the right. For ease of interpretation, the *x*-axis is labeled in reaction-time units (s), although the underlying scale corresponds to promptness (in s^−1^) on a linear axis.

#### Technical Processing Delays

The response times as recorded by the App were expected to be longer than those collected with the Laptop because of the delays introduced by Bluetooth audio streaming and the touchscreen readout. Also, there can be differences in the participant's response speed when hitting a spacebar versus tapping a touchscreen. To align the Laptop and App RTs, we calculated the difference of the median RTs to three well-perceived stimuli to which all participants responded with a low number of outliers for either implementation ([4 Hz, 0.5 c/o], [8 Hz, 0 c/o], and [16 Hz, 0.25 c/o] ripples) (see Supplemental Material S3). We thus obtained a group-level mean delay of 470 ms between the two implementations. All App response times were corrected for this delay.

The variability in the technical processing delays of the device (Laptop and App) should be low for the tests to be reliable. Pilot experiments with normal-hearing participants showed that with the Laptop implementation, differences of only 10 ms in the median RTs to two different stimuli could be reliably reproduced when using 20 trials per stimulus. For the App, this minimum difference was around 20 ms. This indicates that the processing delays in the devices had little variability.

### Statistics

The significance level used in the analysis was 5%. We applied two-sample Kolmogorov–Smirnov tests to determine if two datasets were drawn from the same distribution—specifically, whether the reaction-time distributions from the two measurement blocks (performed for each stimulus in both the App and Laptop implementations) could be combined.

We analyzed the data using two complementary strategies that are appropriate for datasets with substantial numbers of outliers and censored observations (e.g., non-responses), which complicate the use of a standard GLMM (Kruschke, 2015; Lotspeich et al., 2024).

#### Analysis method 1 (used in the main text)

We computed effect sizes using the median RTs and their credible intervals for each condition, based on all available reaction-time data. Credible intervals were obtained using the Estimation Statistics approach described below. This method provides a straightforward and transparent summary of the central tendency and uncertainty, and was therefore chosen as the analysis shown in the main text.

#### Analysis method 2

We also used a Bayesian hierarchical generalized linear model (Kruschke, 2015), which includes the main effects of implementation, participant, and stimulus, as well as all first-order interactions. This approach is identical to that used in Noordanus et al. (2025), where a similarly high proportion of censored reaction-time data was analyzed. This model directly evaluates the effects of implementation, stimulus and participant, as well as their first-order interactions and offers precise posterior estimates and 95% credible intervals for each main effect and interaction. The analysis method and its results are shown in Supplemental Material S4.

#### Estimation statistics

In analysis method 1, we used the MATLAB toolbox (Tumkaya, 2022) of Estimation Statistics (Ho et al., 2019) for statistical comparisons. This method estimates the effect size—the difference between the two distributions being compared—and the lower and upper bound of the 95% credible interval of the effect size by bootstrapping. For this bootstrapping, 5000 samples were taken with replacement. The 95% credible intervals were estimated using the bias-corrected and accelerated bootstrap method, which adjusts not only for bias in the bootstrap distribution, but also for its skewness, thereby improving the accuracy of interval coverage (Efron & Tibshirani, 1994). The reported *p*-value is the likelihood of observing the effect size if the null hypothesis of zero difference is true. Compared to parametric methods, bootstrapping is more robust for data sets with non-normal distributions (Efron, 1987).

### Correlations

Because the reaction-time distributions were markedly skewed (see Figure 2), correlations involving reaction-time measures were calculated using Spearman's rank coefficient, which is less sensitive to outliers than Pearson's *r*. Throughout the manuscript, reported *r* and *p* values therefore refer to Spearman's rank correlations unless explicitly stated otherwise. Statistical significance was defined as rejection of the null hypothesis at the 5% level.

For analyses involving multiple comparisons (e.g., correlations between STM-RT and speech-in-noise thresholds across ripple types), multiplicity correction was applied using the Holm–Bonferroni procedure within each implementation (17 correlations per panel).

In addition to *p* values, we estimated 95% credible intervals of Pearson's *r* computed on inverse RT (promptness), which typically approximates a Gaussian distribution (Noordanus et al., 2025). These intervals were obtained using Bayesian estimation with 500 burn-in iterations and 1,000 Markov Chain Monte Carlo samples and are reported to convey uncertainty in effect size. Correlations were considered to show evidence of association when the 95% credible interval for Pearson's *r* excluded zero.

To examine the relationships among promptness, age, and speech-in-noise perception, we performed a post hoc analysis using multiple linear regression and stepwise regression (MATLAB functions fitlm and stepwiselm, respectively) to identify the optimal linear regression model. The linear regression model was specified as:

(1) $$\begin{array}{c} SRT=\beta_{1}\cdot z(Age)+\beta_{2}\cdot z(Pr)+\beta_{3}\cdot [z(Age)\cdot z(Pr)] \end{array}$$

where *SRT* denotes the 50% speech reception threshold (dependent variable); z(*Age)* is the z-scored age, z(*Pr)* the z-scored median promptness for a selected set of stimuli; and the interaction term tests whether the relationship between age and median promptness depends on age. The coefficients $\beta_{i}$ quantify the relative contribution of each predictor.

Z-scoring was applied to age and promptness to place both predictors on a common scale, facilitating numerical stability in the estimation of the interaction term and enabling direct comparison of effect sizes.

Median promptness was derived either from the three stimuli with the strongest across-participant correlations with SRT—[0 Hz, 0.25 c/o], [8 Hz, 0.5 c/o], and the narrowband stimulus centered at 4 kHz (Figure 8)—or from the three stimuli that elicited reliable responses with few outliers across participants ([4 Hz, 0.5 c/o], [8 Hz, 0 c/o], and [16 Hz, 0.25 c/o]; Supplemental Material S3).

## Results

### Reaction-Time Distributions

Figure 2 shows the reaction-time distributions for a representative selection of 10 participants measured with the App (transparent green) and the Laptop (blue), pooled for all stimuli (excluding the catch stimulus). As can be seen by comparing panels, the distributions varied considerably between participants, both in the location of their maxima and in their shape. In general, however, the distributions of the App and Laptop measurements within participants appeared qualitatively similar. In the following sections, we will quantify these observations.

Before combining the RTs of the two measurement blocks for each implementation in our analysis, we checked for potential differences that could hint at procedural learning effects, or other factors. To that end, we compared the reaction-time distributions for the two blocks, but we found no significant differences for 14/17 stimuli of the App and for 16/17 stimuli of the Laptop measurements (two-sample Kolmogorov–Smirnov test). Because the distributions of the RTs of the two measurement blocks for the four remaining stimuli were largely overlapping, we considered it justified to combine the RTs of the two blocks for the calculation of the median RT for each stimulus.

### Reciprobit Analysis of Reaction-Time Distributions

**Figure 3.**
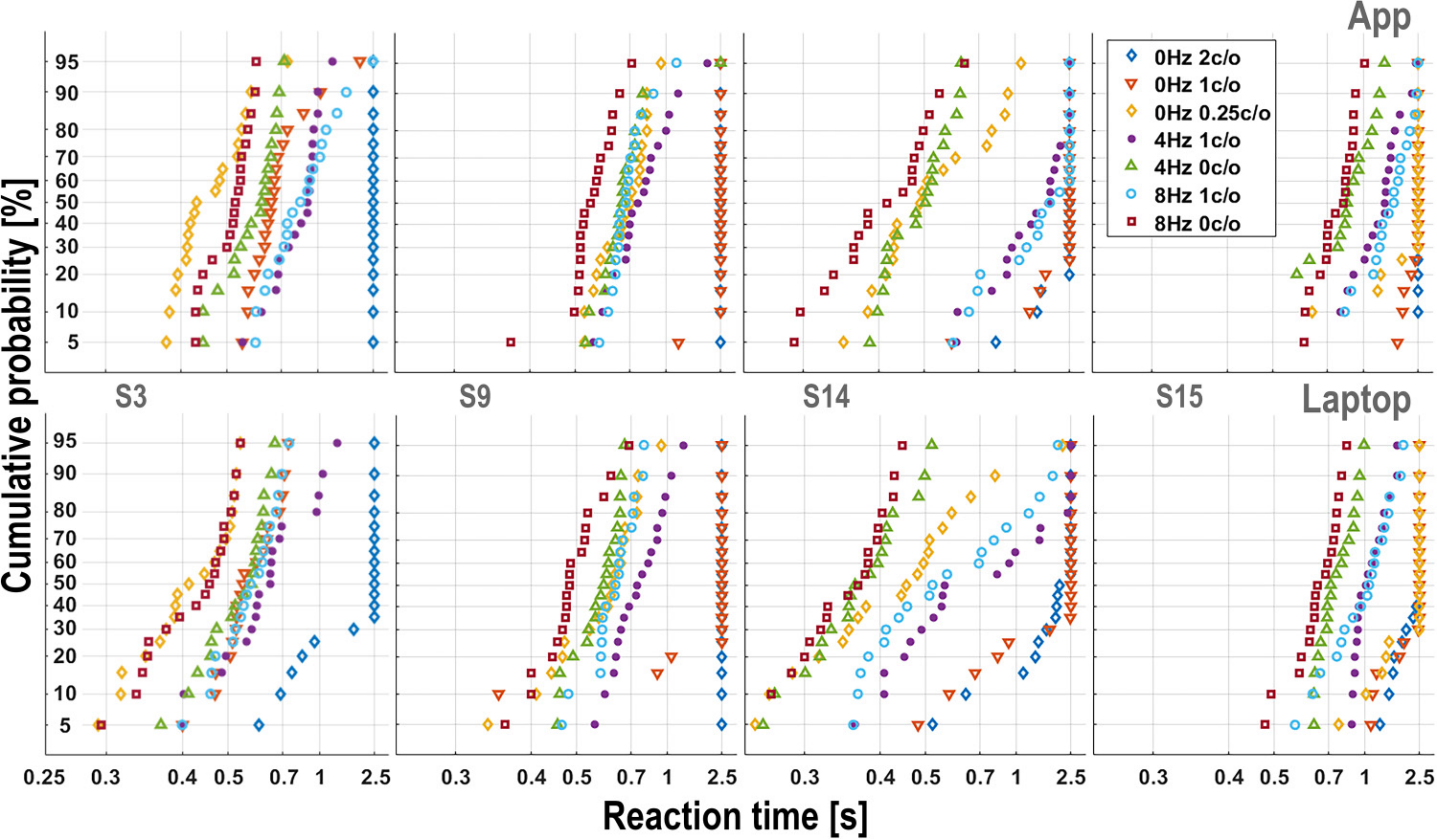
Example reciprobit plots from four participants for seven broadband ripples (see inset in upper-right panel), collected with the App (top row) and the Laptop implementation (bottom row). Cumulative response probability (probit scale) is plotted against promptness (1/RT; *x*-axis labeled in reaction-time units for interpretability). Note the similarity of App and Laptop results, and the large idiosyncratic differences in the reaction times for the different ripples.

To illustrate the response patterns to the different spectrotemporal modulations, Figure 3 shows reciprobit plots (see Methods) for seven selected broadband ripples of four representative participants for the App (top row) and Laptop (bottom) experiments. Participants differed greatly in their response patterns regarding which stimuli were relatively easy (short RTs) or difficult (long RTs, as well as misses, lumped at RT = 2500 ms). Importantly, however, these idiosyncratic patterns were quite comparable for the App versus Laptop implementation within each participant. For instance, the [0 Hz, 0.25 c/o] ripple (yellow diamonds) was easily perceived by S3 for both recording implementations but was nearly inaudible for S15. Also, the stimulus order of the reciprobit plots (from easy, left, to difficult, right) is very similar for the two implementations, but differs markedly for the different participants. These idiosyncratic patterns will be analyzed in more detail below. Reciprobit plots of the four narrowband stimuli are shown in Supplemental Material S5.

Note that the reciprobit plots capture regularities that are not immediately visible in the underlying distribution histograms. For example, lines on the left-hand side correspond to narrowly peaked distributions, while lines with the same slope on the right-hand side correspond to broad distributions.

### Analysis of the Median RTs and App versus Laptop

**Figure 4.**
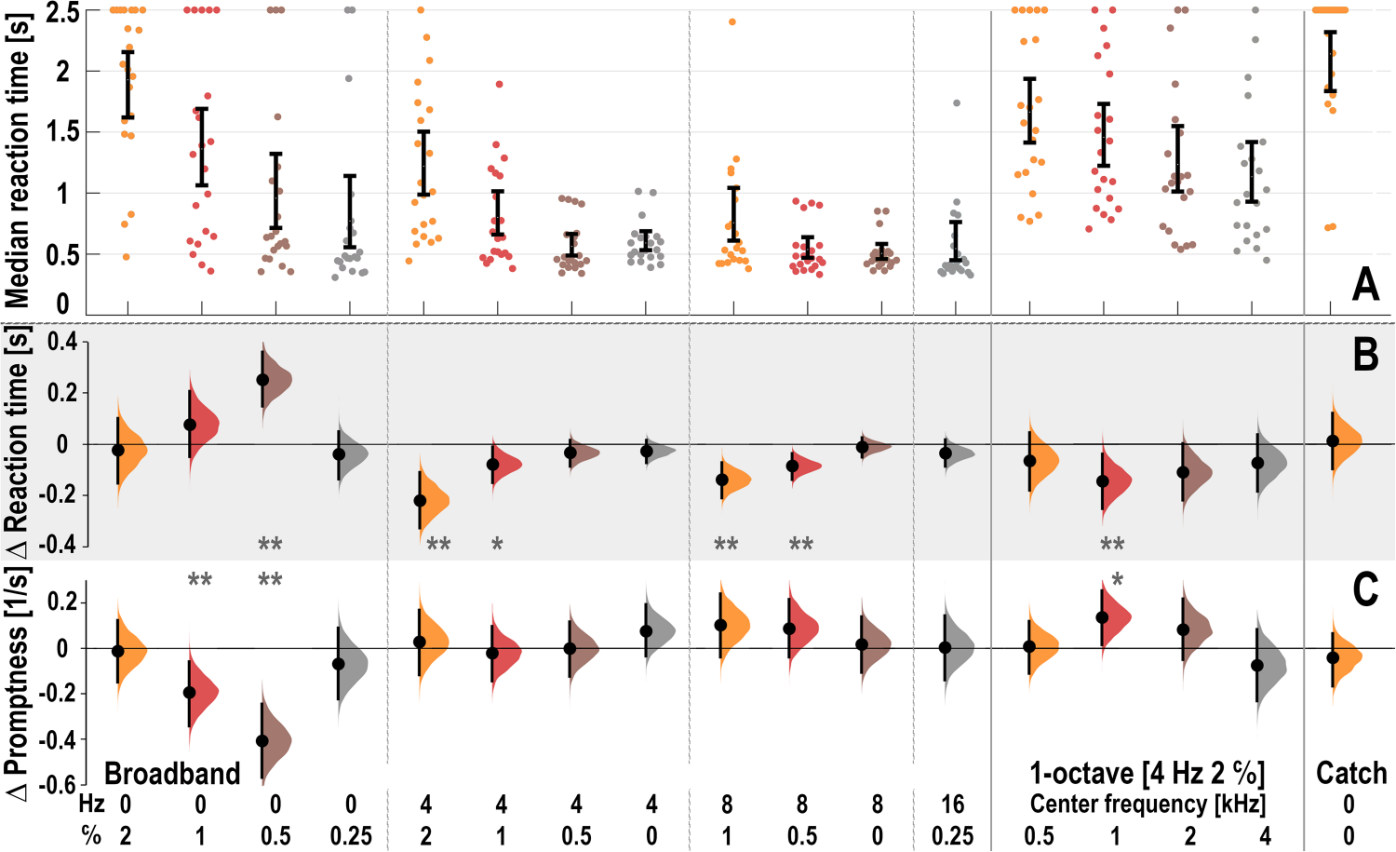
Reaction times and differences between implementations. Stimulus parameters are shown along the abscissa. Vertical error bars indicate 95% credible intervals. (A) Mean of the median reaction times from the App and Laptop measurements. Each dot represents a different participant (S7: App only; S19: Laptop only). (B, C) Distribution of the difference in mean reaction time (B) and promptness (1/RT, C) between the Laptop and App implementations. These differences were calculated using all individual data points, rather than the average of median reaction times per stimulus and participant (as in A). Mean differences are depicted as heavy dots; *: *p* < .05; **: *p* < .01 (not corrected for multiple comparisons across stimuli). Means shifted outward (upward in panel B, downward in panel C) indicate faster App responses relative to Laptop responses (e.g., at [0 Hz, 0.5 c/o]).

**Figure 5.**
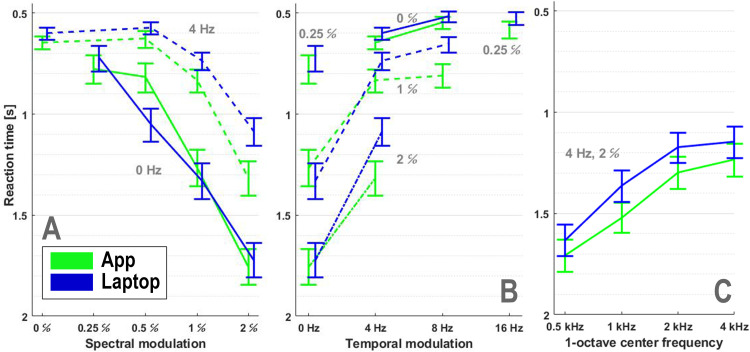
Modulation transfer functions. Spectral (A) and temporal (B) modulation transfer functions based on the means for each broadband stimulus using all responses. Mean reaction times from iso-velocity (0 and 4 Hz; A) and iso-density (0, 0.25, 1 and 2 c/o ripples; B); modulations are connected for visualization purposes (except the 0.25 c/o stimuli). Error bars indicate 95% credible intervals. Note that reaction times are plotted on a reversed scale. Note also that the different iso-stimulus lines are approximately parallel. (C) The narrowband stimuli centered at 2 and 4 kHz elicited mean reaction times comparable to those for the broadband [4 Hz, 2 c/o] ripple. In contrast, responses to the 1 and 0.5 kHz narrowband band ripples were significantly slower (see Discussion).

To analyze differences in RT across participants and stimuli, we calculated, for each ripple condition and each participant, the mean of the median RTs derived from 20 App responses and 20 Laptop responses (Figure 4A). The wide distributions indicate substantial between-participant differences in median RTs for a given stimulus (e.g., [0 Hz, 1 c/o] and, even among the stimuli with shorter RTs, [8 Hz, 1 c/o]). In addition, the magnitude of these between-participant differences varied markedly across stimuli, with some stimuli showing much narrower ranges of median RTs than others. Almost all participants could hear the onset of the broadband temporal modulations (Figure 4A, 4–16 Hz). The broadband [4 Hz, 2 c/o] ripple and the four narrowband ripples (also at 4 Hz, 2 c/o) had comparable median RTs and similar inter-subject variability. The 4 and 2 kHz were the easiest of the narrowband stimuli (see also Figure 5C and Figure S4B in Supplemental Material S4). Although the 500 Hz narrowband stimulus yielded a high median RT of 1750 ms, the response is still clearly different from that to the catch stimulus without modulations.

Supplemental Material S6 shows the median RT to the catch stimulus for both the App and Laptop implementations. Three participants responded relatively frequently to the catch stimulus, with median RTs between 1500 and 2500 ms for both implementations—but substantially later than their responses to actual ripple onsets. Two participants, however, had median RTs of approximately 700 ms for the catch stimulus. These responses were still slower than those elicited by all other stimuli, except for the narrowband 500 Hz ripple (see the reciprobit plot for participant S1 in Supplemental Material S5). Importantly, the catch stimulus elicited the slowest RTs in 19 out of 21 participants.

We found that the median RT per participant for the App and Laptop implementations were reproducible for each stimulus, even though the measurements were performed 2 to 3 months apart. Sometimes, however, a participant reliably detected a particular ripple in the App measurement but failed to do so within 2.5 s in the Laptop measurement, and vice versa. This occurred for 23/323 (7%) of all combinations of stimuli and participants.

To assess the consistency between the two implementations, we computed within-participant Spearman's rank correlations by correlating the stimulus-specific median RTs across implementations separately for each participant. These correlations were significant for all participants, with *r* values ranging from 0.56 to 0.98 (median *r* = 0.89). Because Spearman's rank correlation assesses consistency in ordering, these results demonstrate that the relative difficulty of the stimuli was largely preserved across the two implementations.

To further quantify potential differences between the App and the Laptop implementations, we used all RTs, instead of only their median values (as shown in Figure 4A and in Supplemental Material S7). Figure 4B and C shows the distribution of the difference in RTs between the two implementations for each stimulus across participants, as calculated from the RTs (Figure 4B) and from the promptness (Figure 4C). These differences were obtained by generating bootstrapped distributions (see Methods) of the mean differences between the two implementations, using all measured RTs. Whenever the credible interval of the distribution includes zero, there is no reliable difference between the two response implementations. Significant differences are indicated in Figure 4B (RTs) and Figure 4C (promptness, 1/RT) by one (*p* < .05) or two asterisks (*p* < .01), based on a single uncorrected comparison between App and Laptop. Only the [0 Hz, 0.5 c/o] stimulus showed a large effect size, with *p* < .01 in both analyses, with faster responses in the App implementation than in the Laptop implementation.

The Bayesian analysis model (Methods) further disentangled implementation effects from stimulus effects. Panel D in Figure S4 (Supplemental Material S4) displays the stimulus × implementation interaction, and comparison with the other panels shows that these interaction effects were very small relative to the participant- and stimulus-related effects. Consistent with the main analysis, the [0 Hz, 0.5 c/o] stimulus showed a statistically significant App–Laptop difference, but the absolute effect was modest—approximately 60 ms (about 10% of the mean RT across implementations). Even smaller significant effects (roughly half that magnitude) were observed for another purely spectral ripple—the [0 Hz, 1 c/o] stimulus, which was also significant in the promptness analysis (Figure 4C), again with faster responses for the App—and, in the opposite direction, for the spectrotemporal [8 Hz, 0.5 c/o] and [8 Hz, 1 c/o] stimuli (significant in the reaction-time analysis in Figure 4B).

Note that the widths of the reaction-time difference distributions are stimulus dependent, while the promptness difference distributions are remarkably similar (see Discussion).

In summary, the median RTs per participant collected with the App and Laptop implementations showed close agreement across nearly all ripple conditions, with only one ripple showing a somewhat larger implementation difference, despite the measurements being performed 2 to 3 months apart.

### Modulation Transfer Functions

The auditory MTF characterizes the sensitivity to process spectral, temporal, and spectrotemporal modulations. Typically, these have been determined with threshold measurements. Here we analyze whether similar results can be obtained with RTs to the onset of supra-threshold modulations. Figure 5A and B show the spectral (panel A) and temporal (panel B) MTFs based on the mean reaction-time values for each stimulus. For visualization purposes some stimuli were left out, the joint spectral-temporal sensitivity of the listeners is summarized in Supplemental Material S7. Means were calculated using all RTs across participants, separately for the App (green) and Laptop (blue) measurements. The 95% credible intervals are indicated by the vertical error bars. More precise posterior estimates and 95% credible intervals for the main stimulus effect, derived using the Bayesian analysis model (Methods), are provided in Figure S4B (Supplemental Material S4). The spectral MTF in Figure 5A shows that RTs for stimuli with a temporal modulation of 4 Hz are significantly faster than spectral-only modulations. This is also true for 8 Hz; for example, RTs for [8 Hz, 0.5 c/o] are significantly faster than those for [0 Hz, 0.5 c/o] (Figure 4A), and RTs for [8 Hz, 1 c/o] are significantly faster than those for [0 Hz, 1 c/o] (Figures 4A and 5B). The temporal MTF in Figure 5B shows that the response for 8 Hz is not significantly faster than for 4 Hz. The trend of the spectral MTF across the modulation settings applied is low-pass (i.e., low densities elicit faster responses). The iso-velocity lines in the spectral MTF are roughly parallel, as are the iso-density lines in the temporal MTF. This suggests that time- and frequency sensitivities in the auditory system may arise from largely independent mechanisms, that is, the MTF appears to be approximately separable (Chi et al., 1999; Van der Willigen et al., 2024; Versnel et al., 2009; Veugen et al., 2022).

For comparison with the broadband ripple at [4 Hz, 2 c/o] shown in Figure 5B, the mean RT and credible intervals for the four narrowband stimuli are shown in Figure 5C. The responses to the narrowband stimuli with center frequencies of 2 and 4 kHz are similar to that of the broadband ripple (see also Figure S4B in the Supplemental Material S4).

Overall, detecting a spectral modulation of 0.5 c/o in the absence of temporal modulation is challenging for some participants, whereas a modulation of 2 c/o is very difficult for most participants unless combined with a temporal modulation. Note that the differences between the App and Laptop measurements were small, and that both implementations yielded similar trends.

### Speech in Noise

**Figure 6.**
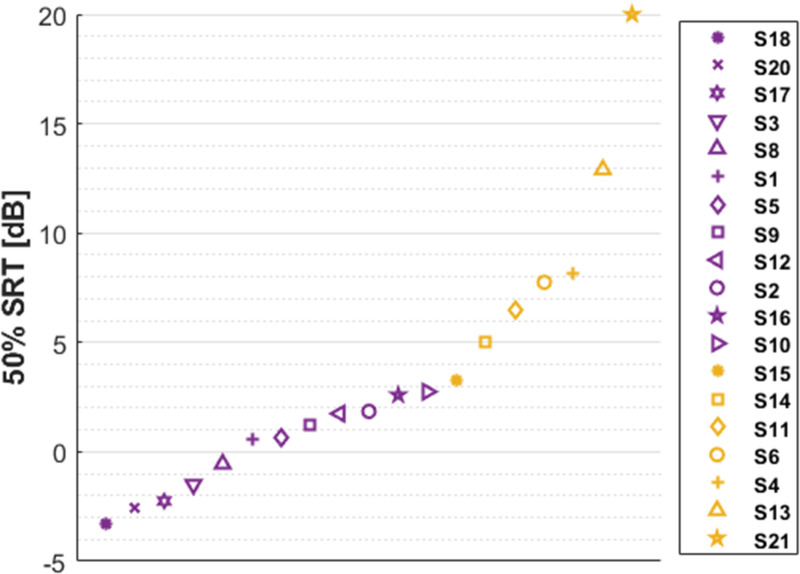
Speech-in-noise thresholds, the mean of two tests. Participants are arranged in ascending order based on their speech-in-noise thresholds. Yellow: medium-poor and poor speech-in-noise performers (Table 1). The 50% SRT of S19 (not shown) exceeded 20 dB (see Methods). SRT: Speech-in-noise threshold (50%).

Figure 6 shows the means of two free-field speech-in-noise tests for each participant, ordered by ascending speech-in-noise threshold. A higher threshold indicates poorer speech-in-noise perception performance. Medium-poor and poor speech-in-noise performers are shown in yellow, following the classification in Table 1.

### Correlation Between STM-RT and Speech in Noise

To address our third aim—verifying the relationship between reaction-time-measured spectrotemporal modulation sensitivity (a low-level auditory processing measure) and speech-in-noise perception (a higher-level auditory outcome)—we calculated Spearman's rank correlations between STM-RTs and speech-in-noise thresholds for each ripple type.

**Figure 7.**
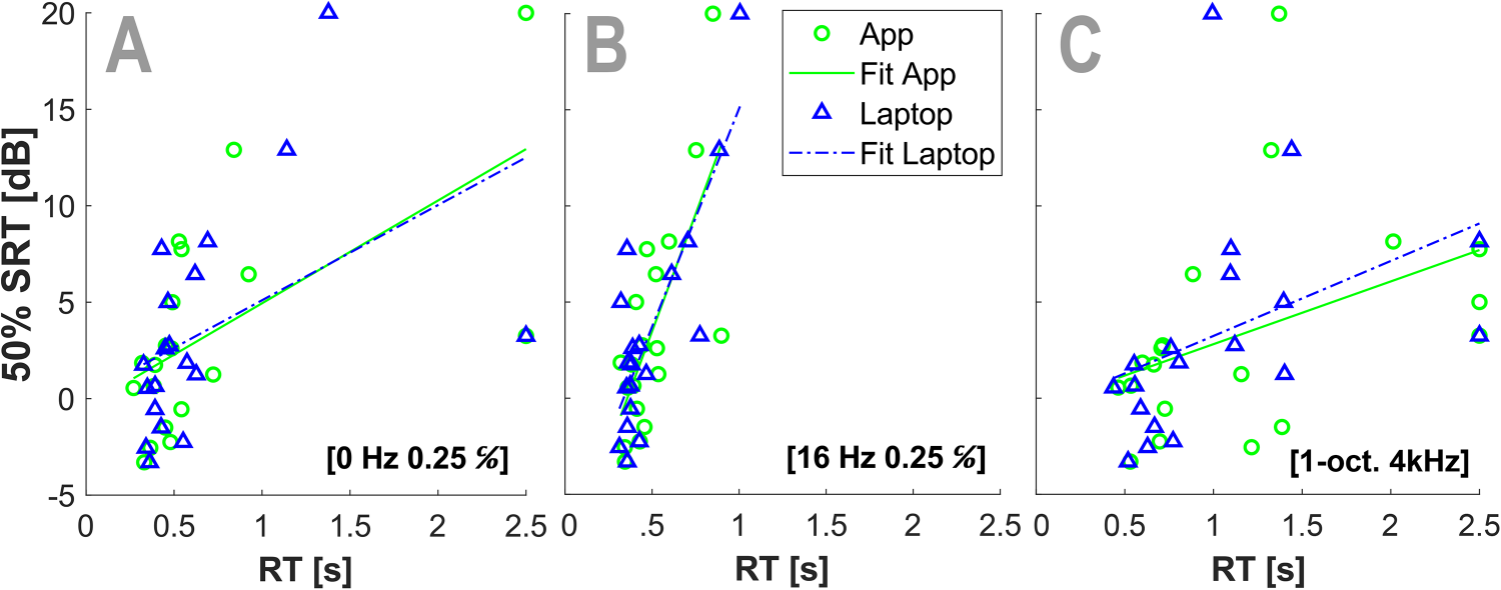
Examples of speech perception versus reaction time for the (A) [0 Hz, 0.25 c/o], (B) [16 Hz, 0.25 c/o], and (C) [1-oct 4 kHz] stimuli for App (green) and Laptop (blue). These three stimuli showed a strong correlation between speech perception and reaction time. Each symbol represents the means for a single participant. The displayed lines represent linear fits. Note that the linear fit in panel A is strongly influenced by the two participants that could not detect the stimulus (see also Figure 4A). However, the influence in the calculation of the correlation of these responses is limited, since we used Spearman's rank to calculate the *r* (see Methods).

Figure 7 illustrates this relationship for three ripple conditions that showed relatively strong associations, shown for both the App and Laptop implementations.

**Figure 8.**
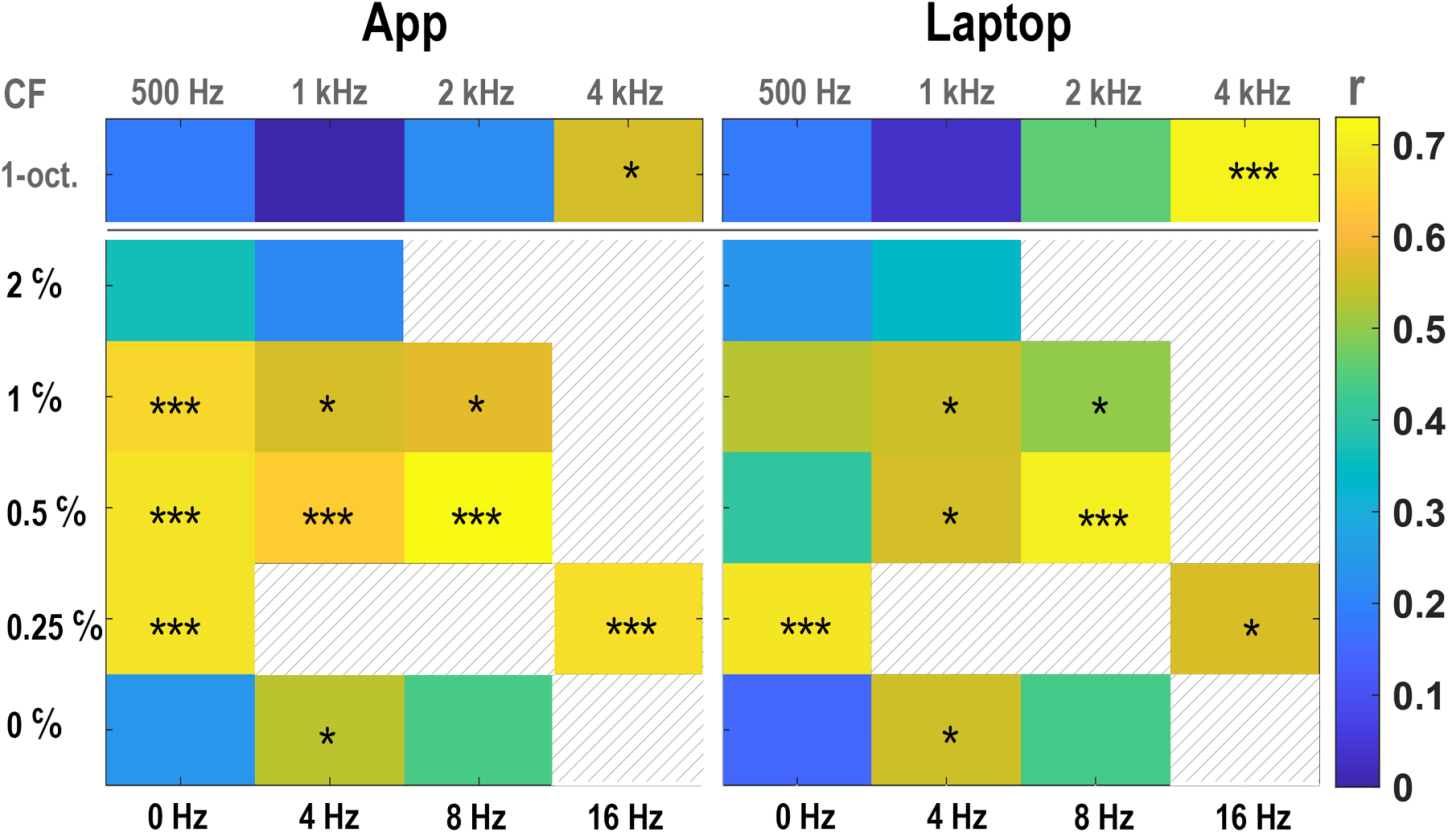
Correlation between STM-RT and speech-in-noise perception. Spearman’s rank correlation, *r*, between STM-RT and speech-in-noise perception, analyzed for the App (left panel) and Laptop data (right panel). The top row of colored boxes shows the correlations for the narrowband [4 Hz, 2 c/o] stimuli (the center frequency CF of the 1-octave band is indicated above). The other five rows show the correlations for the broadband stimuli (stimulus order is the same as in Table 2). Maximum of the color bar: *r* = .73. Asterisks indicate correlations whose 95% credible interval excluded zero. *** Further indicates significance after Holm–Bonferroni correction within each panel (17 tests).

The results obtained for all stimuli are summarized in Figure 8 for the App (left) and Laptop (right) implementations. Because multiple ripple types were tested within each implementation, multiplicity correction was applied using the Holm–Bonferroni procedure (17 correlations per panel; see Methods). In Figure 8, correlations whose 95% credible interval excluded zero are marked with asterisks, and three asterisks (***) indicate correlations that remained significant after Holm–Bonferroni correction.

Correlations between STM-RTs and speech-in-noise perception were highly consistent across the App and Laptop implementations, even though the measurements were performed in different sessions 2 to 3 months apart. Of the 17 stimuli, the same eight stimuli showed evidence of association in both implementations (i.e., 95% credible interval excluding zero).

Implementation-specific differences were limited to two purely spectral stimuli ([0 Hz, 0.5 c/o] and [0 Hz, 1 c/o]), which showed significant correlations for the App but not for the Laptop implementation. Notably, for the App these correlations also survived Holm–Bonferroni correction. No ripple type showed the reverse pattern (i.e., significant for Laptop but not for App). Overall, the correlation was slightly higher for the App, where the strongest correlations were obtained for six ripples (***), with coefficients of determination in the range *r*^2^ = 0.41–0.53.

Of the broadband stimuli, only the 2 c/o stimuli and the purely temporal 8 Hz had no significant correlation with speech in noise. As described in the Introduction, this finding is in line with Elliott and Theunissen (2009), who reported that speech is best represented by spectral modulations < 1.0 cycles/octave and ripple velocities below 10 Hz. Of the narrowband [4 Hz, 2 c/o] stimuli, only the ripple with center frequency 4 kHz correlated significantly with the speech-in-noise reception thresholds.

As a robustness check, we repeated the correlation analysis for the App RTs using speech-in-noise thresholds obtained with the App instead of the Laptop. The resulting pattern of correlations was highly similar to that shown in Figure 8 (see Supplemental Material S8).

To obtain more stable participant-specific estimates of RTs and speech-in-noise thresholds, we additionally averaged the App and Laptop measurements within participants. Using these combined estimates yielded overall slightly stronger correlations between STM-RT and speech-in-noise performance, and a greater number of ripple types remained significant after Holm–Bonferroni correction. Full details are provided in Supplemental Material S8.

Taken together, speech-in-noise perception was best predicted by the RTs to the 0.25 cycles/octave broadband ripple without temporal modulation and the 0.5 cycles/octave broadband ripple modulated at 8 Hz. Of the narrowband bandpass filtered ripples, the STM-RT to the 4 K band ripple best predicted speech-in-noise perception.

## Discussion

### Summary

We explored whether RTs to an unpredictable change in an acoustic stimulus provide a reliable assessment of the spectrotemporal sensitivity of CI users. To that end, 20 unilateral CI users completed two different experimental protocols, separated by 2–3 months. With the App implementation, the spectrotemporal ripples were presented over a Bluetooth connection to the CI processor, and listeners touched the smartphone screen as soon as they detected the ripple onset. The Laptop implementation was performed under free-field conditions and participants pressed a computer's spacebar instead of a touch screen. We also measured speech-in-noise perception.

We analyzed the reaction-time distributions for the different ripples by constructing reciprobit plots (Methods, Supplemental Material Figure S1B and C). Our data show that in most cases the results could be quantified by near-linear relationships (Figure 3), in line with the LATER model (Supplemental Material S1). As a consequence, false positive responses, not triggered by the ripple onset, and misses (stimulus not perceived), could be readily identified as outliers.

To illustrate that the STM-RT method provides a valid measure for spectrotemporal sensitivity, we constructed the spectral and temporal MTFs (Figure 5), which revealed close resemblance to what is known from the literature (Chi et al., 1999; Elhilali et al., 2003; Elliott & Theunissen, 2009; Shannon, 1992; Van der Willigen et al., 2024). Another indication of the reliability of the RT paradigm is that results from the App and Laptop implementations were reproducible, with the reaction-time distributions varying from ripple to ripple in an idiosyncratic way (e.g., Figures 2 to 4), even though the measurement sessions were scheduled 2 to 3 months apart.

RTs correlated with the speech-in-noise reception threshold for most of the ripples used in our experiments, with shorter RTs corresponding to better speech performance (Figures 7 and 8).

Taken together, our results demonstrate the feasibility and reliability of the ripple reaction-time paradigm to collect large amounts of data on spectrotemporal sensitivity from CI users using the app under controlled conditions, supporting its potential for future use in home environments. Such data might eventually be used to optimize CI fittings and may thus help to reduce the large variability in speech-in-noise perception performance among CI users (Blamey et al., 2013; Pisoni et al., 2017).

In what follows, we will discuss how to optimally use the reaction-time paradigm in psychophysical measurements. We will also discuss the correlation with speech in noise and limitations of the current study.

### Measuring RTs

An advantage for the reaction-time paradigm over measuring the modulation detection threshold for each ST ripple is that modulations can be presented well above the detection threshold, with the RT reflecting the task's difficulty. Another advantage is that detecting a ripple modulation onset presented within an ongoing noise carrier is more representative for processing natural speech and music than comparing distinct stimuli, separated by silence, as in an AFC task (Zhang et al., 2019).

The goal of our experiments was to assess spectrotemporal sensitivity with as little intrusion of other processes as possible. If the participant reacts as fast as possible to the target, the response can be considered predominantly stimulus driven with little interference from non-acoustic factors such as working memory and linguistic mechanisms. Due to impoverished input caused by physiological reasons (e.g., the onset and duration of deafness) and/or device-related issues, the time required for stimulus identification is prolonged in many hearing-impaired listeners compared to normal hearing (Burkholder & Pisoni, 2003; Veugen, 2017). For difficult stimuli, for example, vocoded sounds, also normal-hearing listeners have prolonged responses (Veugen et al., 2022).

Within the LATER model (Supplemental Material S1), RT is explained by four factors: the strength of the neural signal (the evidence rate), internal processing noise, prior knowledge, and the decision threshold. Also factors such as hearing impairment, health conditions, and aging affect RTs and are incorporated in one or more of these parameters. All parameters may vary across listeners, contributing to inter-subject differences in overall RT.

Note that prior knowledge and decision criterion reflect cognitive mechanisms and should be kept as constant as possible throughout the experiments, as this enables a valid comparison of responses to different stimuli. This ensures that observed differences in RT between stimuli primarily reflect variations in evidence rate and internal processing noise, thereby providing a more direct measure of spectral and temporal sensitivity.

In addition, several experimental design factors are important for an adequate assessment of stimulus-evoked RTs. These factors—unpredictability, training, stimulus quality and measurement accuracy—are discussed in Supplemental Material S9.

RTs reflect sensitivity to a change in the acoustic input and can probe different auditory processes when the stimulus set is designed accordingly. Here, we aimed to quantify sensitivity to well-defined spectrotemporal parameters by comparing performance across systematically varied stimulus conditions.

In this context, one may wonder whether listeners could have relied primarily on local fluctuations within a single spectral region. Although we did not optimize the stimulus set to answer this question, the results suggest that this is unlikely. Performance with the 4 kHz narrowband stimulus matched that of the broadband ripple, suggesting that listeners may have used whichever frequency region provided the most salient cue rather than fluctuations confined to a single narrow band. Additional evidence against exclusive reliance on local fluctuations comes from the finding that RTs were often shorter for combined spectrotemporal modulations than for the corresponding single-dimension conditions (e.g., [4 Hz, 0.5 c/o] vs. [0 Hz, 0.5 c/o] or [4 Hz, 0 c/o]; Figure 4A; Supplemental Material S4, Figure S4_1B). Such patterns may reflect statistical facilitation, as predicted by race-model accounts (Colonius, 1990): if listeners monitor multiple channels and detection is governed by the fastest one, combined conditions yield shorter responses purely for probabilistic reasons—implying monitoring beyond a single local cue. Alternatively, shorter RTs may arise from genuine integrative processing across frequency and time if they violate the race-model prediction.

To further examine possible differences in local fluctuations across stimuli, we simulated electrodogram outputs using the Generic MATLAB Toolbox (GMT) from Advanced Bionics (Supplemental Material S10). A local-cue might potentially explain faster RTs at the most basal electrode (electrode 16) for the more distinct modulation patterns for the [4 Hz, 0.5 c/o] compared to [4 Hz, 0 c/o] (Figure S10_1). However, our previous work using direct electrode stimulation in the same cohort (Noordanus et al., 2025) demonstrated substantially lower sensitivity on this most basal electrode to changes in modulation frequency. This makes it unlikely that a local cue at this electrode drives the observed RT differences. Moreover, if electrode 16 dominated detection, the broadband [4 Hz, 2 c/o] stimulus (Figure S10_1D) should have elicited slower responses than its 4-kHz narrowband counterpart—where the modulation at electrode 16 is more pronounced (Figure S10_2A). However, this was not observed.

### App versus Laptop Implementations: Comparisons and Limitations

The patterns of the reciprobit lines for the various spectrotemporal ripples, although very different between participants, were reproducible for the App versus Laptop implementations, even though measurements were taken months apart (see Figure 3).

The difference distributions for RT between App and Laptop were highly stimulus dependent (Figure 4B). In contrast, the widths of the difference distributions for promptness were very similar (Figure 4C). These observations cannot be explained by a stimulus-independent trial-to-trial variability in the technical delay of the App, which would have resulted in reaction-time difference distributions with similar widths in Figure 4B. Rather, the observed invariance of the promptness results follows from the remarkable similarity of the median and the slope of the reciprobit plots for the different stimuli across the two implementations (Figure 3). In the LATER model, these slopes reflect the internal processing noise (Supplemental Material, Figure S1C). Our data therefore indicate that internal processing noise remained largely stable across stimuli and sessions.

Overall, response-time differences between stimuli for App vs. Laptop implementation were close to zero after correction for a fixed delay (see the section “Technical delays” below), as shown by the very small implementation × stimulus interaction (Figure 4D, Supplemental Material S4). The largest effect was observed for the purely spectral [0 Hz, 0.5 c/o] ripple, for which responses were consistently faster in the App implementation (Figure 4B,C). The likewise purely spectral [0 Hz, 1 c/o] ripple showed a smaller but still significant difference in the same direction, whereas two spectrotemporal 8 Hz stimuli exhibited slightly faster responses in the Laptop condition.

Because all participants completed the App implementation first and the Laptop implementation approximately 3 months later, a potential order effect related to task learning cannot be fully excluded. Such an effect would be expected to result in generally faster RTs in the second session, favoring the Laptop condition. However, it is questionable whether substantial task learning would persist after such a long interval, particularly given the procedural and implementation differences between sessions. Moreover, learning-related effects are unlikely to manifest as a fixed offset across stimuli of varying difficulty.

Taken together, these patterns suggest that subtle differences in the actual acoustic delivery of the two implementations, rather than systematic learning effects or latency correction procedures, may have contributed to the small observed effects.

***Technical delays.*** As argued above, for a valid comparison of RTs collected with different implementations across different labs, it is important to correct for technical delays in the signal processing pathway as much as possible. Since we did not have direct access to the internal clocking of smartphone data processing and Bluetooth transmission, we estimated the extra delay for the App relative to the Laptop implementation by comparing the results for three well-perceived stimuli (see Supplemental Material S3). For future applications, it is recommended to electronically assess the true technical delays between stimulus onset and CI-electrode activation, and between actual screen touch and its associated timestamp.

***AGC activation and responses to catch trials.*** In the current experiments, the AGC was activated approximately 400 ms after the onset of the static pink noise, resulting in a sudden level reduction (Figure 1). Most participants did not respond to this change, as indicated by the small fraction of RTs under 140 ms relative to ripple onset (App: 1%; Laptop: 0.7%). Three participants did, however, respond relatively often to the catch stimulus (Supplemental Material S6), although markedly later than to stimuli containing an actual ripple onset. This indicates that responses to genuine ripple onsets were true acoustic change detections rather than anticipatory responses. Panel B of Figure S4 (Supplemental Material S4) further shows that the catch stimulus elicited the slowest responses across all stimuli.

Two participants produced many false-positive reactions to the catch stimulus (example in Supplemental Material S5, see also S6). Because their responses to the narrowband 500 Hz ripple were still slower than their responses to the catch stimulus, these appear to reflect genuine responses of a perceived change rather than predictive responses. In these cases, the cue eliciting a response was likely less salient than the onset of an audible modulation and may therefore, on a speculative basis, have relied on gradual changes in perceived level. Accordingly, responses may have been triggered either by an AGC-induced level change associated with stimulus onset or by a subtle perceptual change related to the nominal “modulation” onset. However, given the current data—obtained with a modulation-onset randomization window of only 0.5 s—we cannot distinguish between these possibilities with certainty.

### Modulation Transfer Functions

We compared the MTFs from Figure 5, based on RTs to the onset of supra-threshold modulations, with the results of Zheng et al. (2017) for CI users, which were based on just-noticeable-differences between modulated and unmodulated sounds. For spectral-only modulation, both RTs and modulation detection thresholds increased as spectral modulation increased. Zheng et al. (2017) did not find significant differences between iso-velocity lines of 8, 16, and 32 Hz. Similarly, we did not find significant differences between the 4 and 8 Hz conditions. However, RTs were significantly faster for joint spectrotemporal modulations compared to spectral-only modulations (Figures 4A and 5B). In contrast, the results of Zheng et al. (2017) did not reach statistical significance when comparing modulation detection thresholds for purely spectral stimuli and their spectrotemporal counterparts (their Figure 2C). Nevertheless, in an overall analysis, they reported a significant improvement for joint spectrotemporal modulations relative to predictions based on the measured effects of spectral- and temporal-modulation-only conditions.

For the narrowband stimuli, we observed a trend toward faster RTs as the center frequency increased from low to high (Figure 5C). A post-hoc analysis using the GMT toolbox (Supplemental Material S10) provided by Advanced Bionics to simulate CI electrode stimulation patterns suggested that the pulse patterns encoding the imposed modulations were more distinct in the higher-frequency bands than in the lower-frequency ones (see Supplemental Material S10, Figure S10_2). This was also visible in the broadband stimuli (Figure S10_1D). A comparable frequency-dependent effect was reported by Geys, Sijgers, Oesch, Chalupper, et al. (2026) in CI users fitted with an Advanced Bionics Marvel processor, with poorer performance around 500 Hz than in bands with higher center frequencies using 2-octave rather than 1-octave stimuli, further supporting the generality of this pattern across bandwidths.

Three factors may contribute to the reduced distinctness at low frequencies. First, in the lower-frequency channels, part of the modulation trough could fall below audibility for participants with higher aided thresholds. However, this is not expected to produce a systematic effect across participants. Second, the pre-emphasis filtering applied by the CI processor attenuates lower frequencies; for example, a 500 Hz tone is attenuated by approximately 10 dB relative to a 4 kHz tone. Third, the analysis filters in the lower-frequency channels are narrower, which may produce slower and more pronounced inherent envelope fluctuations in band-limited noise, which can mask or compete with the imposed modulations, thereby reducing its salience.

Simulations by Geys, Sijgers, Oesch, Chalupper, et al. (2026) using the GMT toolbox indicate that the pre-emphasis filter has only a minor effect on the electrodogram output. Increasing spectral resolution in the apical channels (via more FFT bins) also did not improve modulation coding, whereas increasing the bandwidth of these channels did yield a clearer representation of the imposed modulation, with the improvement occurring slightly basal to the most apical channels but still in the apical direction. This supports the interpretation that narrower low-frequency filters—and the slower inherent envelope fluctuations they produce—can obscure the imposed modulation in apical channels.

A similar frequency-dependent pattern was observed in a normal-hearing control group (data not shown), with slower detection for low-frequency narrowband stimuli. This suggests that the effect may partly reflect inherent auditory-filter characteristics—narrower filters at low frequencies produce slower envelope fluctuations (Dau et al., 1999; Varnet & Lorenzi, 2022). These characteristics reduce the salience of modulation onset independently of CI processing.

### Effects of Age on RTs and Speech-in-Noise Perception

In our dataset, speech-in-noise perception showed a significant association with age (Pearson's *r* = .60, *p* = .0071), consistent with the literature (Jin et al., 2014). Although RTs for 10 (App) and 8 (Laptop) of the 16 ripples correlated significantly with speech-in-noise perception performance (Figure 8), their association with age did not reach significance. This was true both when using the median promptness per participant across all ripples (Pearson's *r* = –.32; 95% credible interval: −0.66 to 0.09) and when analyzing each ripple separately. The two stimuli with RTs closest to a significant effect ([16 Hz, 0.25 c/o] and [0 Hz, 0.5 c/o]) also remained non-significant (*r* = −.40; 95% credible interval: −0.71 to 0.04 and *r* = −.37; 95% credible interval: −0.70 to 0.05, respectively, based on median promptness from the combined laptop and app measurements). These findings suggest that, in this cohort, age-related differences in speech-in-noise perception were not reflected in reaction-time performance. It is worth noting that age-related slowing in a congruent single-task paradigm such as ours typically amounts to less than 200 ms (Tun & Lachman, 2009), which is substantially smaller than the between-participant differences observed here (Figure 4A).

To examine this further, we identified the optimal linear regression model (Equation 1) using age and the median promptness across the three stimuli showing the highest correlations with speech-in-noise perception performance ([0 Hz, 0.25 c/o], [8 Hz, 0.5 c/o], and narrowband 4 kHz [4 Hz, 2 c/o]; Figure 8). In this analysis, the optimal model included promptness as the sole predictor.

We repeated this procedure for the three well-perceived stimuli to which all participants responded with few outliers for either implementation ([4 Hz, 0.5 c/o], [8 Hz, 0 c/o], and [16 Hz, 0.25 c/o]; Methods). In this case, the optimal model included age, median promptness, and their interaction. The interaction term had the largest (and negative) coefficient, indicating that the effect of spectrotemporal sensitivity on speech-in-noise performance outweighed the direct contribution of age alone. Overall, this model accounted for 74% of the variance in speech-in-noise thresholds. Table 3 summarizes the results.

**Table 3.** Optimal Linear Regression Models (Eqn. 1) Using Age and Median Promptness.

	Using Median Promptness Across the Three Stimuli With the Highest Correlation With Speech		Using Median Promptness Across the Three Well-Perceived Stimuli	
	**Adjusted R^2^: 0.44 (*p*: .0012)**		**Adjusted R^2^: 0.74 (*p*: .00010)**	
Parameter	Coefficient (β)	*p*	Coefficient (β)	*p*
Median promptness	−0.69	.0012	−0.23	.21
Age			0.49	.0046
Median promptness × age			−0.59	.010

The left panel shows results for the three stimuli with the strongest correlations with speech-in-noise performance ([0 Hz, 0.25 c/o], [8 Hz, 0.5 c/o], and narrowband 4 kHz [4 Hz, 2 c/o]). The right panel shows results for the three well-perceived stimuli ([4 Hz, 0.5 c/o], [8 Hz, 0 c/o], and [16 Hz, 0.25 c/o]).

Taken together, these analyses suggest that spectrotemporal sensitivity accounts for approximately 50% of the variance in speech-in-noise performance, with age-related cognitive factors contributing an additional ∼25%.

### Correlation With Speech in Noise

Short RTs to the ripple onsets correlated with good speech-in-noise perception, especially for purely spectral ripples (up to 1 cycles/octave), and combined spectrotemporal ripples between 4–16 Hz and 0.25–1.0 cycles/octave (Figures 7 and 8).

Since our stimulus set was selected for its expected relation to speech-in-noise perception, the mean RTs carried predictive value for speech-in-noise performance. Note that the observed across-participant correlations cannot be explained by a general tendency for poorer speech performers to respond more slowly. In that case, the purely temporal 8-Hz ripple—which does not correlate significantly with speech-in-noise perception (Figure 8)—would also show such an association, which it did not.

In addition, Figure 4 and the participant × stimulus interaction in Figure S4E (Supplemental Material S4) show pronounced within-participant variation across stimuli, with effect sizes comparable to the main effects of stimulus and participant. This demonstrates that listeners do not differ merely by a uniform shift in RT, but rather exhibit distinct sensitivity patterns across ripple types. Consistent with this, Figure 8 reveals substantial differences between ripple conditions in the strength of their correlations with SRT. Such differential correlations can only arise when participants vary across stimuli in a stimulus-specific manner, rather than through a simple overall speeding or slowing across tasks.

All 12 good and medium speech-in-noise performers (Table 1) showed their fastest responses to the [0 Hz, 0.25 c/o] or the [16 Hz, 0.25 c/o] ripple, and the RTs for these spectral and spectrotemporal ripples also correlated strongly with speech in noise perception (Figures 7A, B and 8; see also Supplemental Material S10, Figure S10_3 for electrodogram examples). In contrast, 4/7 medium poor and poor speech-in-noise performers did not exhibit their fastest responses for these stimuli. Instead, this group responded fastest to the purely temporal [8 Hz, 0 c/o] ripple, which did not correlate significantly with speech-in-noise perception (Figure 8). This result suggests that these poorer performers did not benefit from the immediately available spectral cues, but instead relied predominantly on temporal cues alone. This is consistent with the idea that speech-in-noise perception in CI users benefits from combining spectral and temporal modulation information (Zheng et al., 2017, see also above).

#### Narrowband Stimuli

Stimuli whose RTs correlated with speech-in-noise perception generally elicited relatively short RTs. A notable exception was the narrowband 4 kHz ripple: although its RTs showed a strong correlation with speech-in-noise perception (Figures 7C and 8), the mean RT exceeded 1 s and exhibited substantial variability across listeners (Figures 4A and 7C). The strong association between RT for this 4 kHz narrowband stimulus and speech-in-noise perception aligns with findings reported for unaided hearing-impaired listeners (Mehraei et al., 2014). As discussed above, higher-frequency narrowband stimuli are encoded more distinctly in CI processors and, potentially, also in normal or impaired acoustic hearing owing to auditory-filter characteristics, likely resulting in a higher effective SNR of the imposed modulation. This increased salience could explain why a high-frequency narrowband stimulus can yield long absolute RTs, yet still correlate strongly with speech-in-noise performance.

Interestingly, in our direct electrode-stimulation study with the same cohort (Noordanus et al., 2025), the strongest correlations with SRT were found for electrodes corresponding to the 2-kHz region, again suggesting that sensitivity at higher frequencies may be particularly predictive of speech-in-noise outcomes.

### Clinical Implications

Used as an at-home tool, ripple reaction-times will enable frequent monitoring by the clinician of the CI user's spectrotemporal sensitivity over time, which can be used to decide whether a visit to the clinic may be necessary. After implementing the aforementioned improvements, a pilot study on at-home use will be required. The App is designed to be user-friendly for both patients and clinicians, with data instantly accessible to clinicians via a cloud-based platform. In a future update, clinicians will have the option to either use the default settings or manually configure the specific stimuli to be measured. The laptop implementation is well-suited for research purposes and can be readily made compatible with implants from other manufacturers.

Given the across-participant correlations between RTs for specific ripples and speech-in-noise performance, these measures may offer predictive value shortly after first switch-on, providing early insight into expected speech outcomes. In addition, ripple RTs provide a rich source of information on listening effort and may serve as a valuable alternative for speech-based paradigms (Gagné et al., 2017) and the highly variable pupil-dilation method to assess (low-level) aspects of listening effort of CI users (Prodi & Visentin, 2022; Zekveld et al., 2018).

Assuming that changes in speech processor fitting immediately affect individual spectrotemporal sensitivity and that this sensitivity (in contrast to speech perception) changes relatively little with experience after the fitting session, ripple reaction-times to a set of ripples relevant for speech-in-noise perception (Figures 7 and 8) may, in principle, provide an immediate measure of fitting-related changes within individual participants, without having to acclimatize to the new settings. In our future work, we will test whether these measures can be used to guide fitting decisions in routine practice (Van Opstal & Noordanus, 2023).

The STM-RT method may enable detailed assessment of spectrotemporal sensitivity with high spectral and temporal resolution and may contribute to the refinement of CI fitting strategies. Including narrowband ripple stimuli to selectively probe the frequency range relevant for speech perception could be beneficial for obtaining frequency-specific measures, such as guiding targeted adjustments to frequency mapping in the CI processor. However, the development of such applications—particularly those relying on carefully selected narrowband stimuli expected to be especially informative—will require careful consideration of the systematic center-frequency-dependent effects observed here, with RTs tending to decrease from low to high center frequencies.

Ripple reaction-time data collected before and after fitting may be used to train future machine learning algorithms aimed at supporting individualized CI fitting. The goal would be to identify parameter configurations that yield shorter RTs, reflecting higher spectrotemporal evidence rates (Van Opstal & Noordanus, 2023). Recent work has demonstrated that reaction-time paradigms can also be applied at the level of individual electrodes, using direct electrode stimulation to assess detection of changes in modulation frequency or electrode location in the presence of a masker (Geys, Sijgers, Oesch, Boyle, et al., 2026; Noordanus et al., 2025). Such measurements allow estimation of information-transfer fidelity along the electrode array and through central auditory pathways, providing insight into the spectrotemporal resolution that could potentially be achieved through tailored fitting strategies. These data may guide frequency allocation across electrodes.

In a subsequent step, the STM-RT test could be used to evaluate how alternative fittings and programming strategies affect sensitivity to speech-relevant spectrotemporal modulations. To support this development, further research is required in two main directions. First, RTs should be examined across a broader range of spectrotemporal ripple conditions before and after fitting adjustments. Second, it needs to be established that fitting-related changes can be evaluated acutely using RTs to specific ripples, and that such changes reliably correspond to improvements in speech-in-noise perception after adaptation.

### Conclusions

Ripple reaction-times revealed systematic differences in the sensory-neural processing speed for different spectrotemporal modulations that are relevant for speech perception of CI users. The differences between stimulus-evoked responses were idiosyncratic, robust between implementations, and consistent over time. These results demonstrate fundamental characteristics of spectrotemporal sensitivity for individual participants. The robust response variation between stimuli and participants provides a foundation for future work aimed at developing machine-learning algorithms that can exploit these data before and after fitting to optimize speech perception.

The App implementation is convenient for clinical use and provides a promising basis for future at-home applications, which may enable closed-loop evaluation and improvement of CI fitting feasible in practice. This approach may ultimately help to reduce the large variability in speech-in-noise perception performance among CI users.

## Supplemental Material

sj-docx-1-tia-10.1177_23312165261436198 - Supplemental material for Measuring Spectrotemporal Sensitivity in Cochlear Implant Users With a Reaction-Time Paradigm: A Comparison of Two Implementations Supplemental material, sj-docx-1-tia-10.1177_23312165261436198 for Measuring Spectrotemporal Sensitivity in Cochlear Implant Users With a Reaction-Time Paradigm: A Comparison of Two Implementations by Elisabeth Noordanus, Lucas H.M. Mens, Josef Chalupper, Tobias Balkenhol, Marc M. Van Wanrooij and Adrianus John Van Opstal in Trends in Hearing

## Abbreviations

1-oct xxxx: One octave bandwidth centered at xxxx (0.5/1/2/4) kHz
AFC: Alternative forced choice
AGC: Automatic gain control
CI: Cochlear implant
c/o: Cycles/octave (spectral modulation, “density”)
MTF: Modulation transfer function
RT: Reaction time
SNR: Signal-to-noise ratio
SRT: Speech reception threshold
STM: Spectrotemporal modulation
STM-RT: Reaction time to the onset of a spectrotemporal modulation
