# Supplementary material for: Measuring Spectrotemporal Sensitivity in Cochlear Implant Users With a Reaction-Time Paradigm: A Comparison of Two Implementations
Source: Trends Hear. 2026 Mar 30;30:23312165261436198. doi: 10.1177/23312165261436198 (PMC13039611; doi:10.1177/23312165261436198)
Supplement: sj-docx-1-tia-10.1177_23312165261436198 - Supplemental material for Measuring Spectrotemporal Sensitivity in Cochlear Implant Users With a Reaction-Time Paradigm: A Comparison of Two Implementations [file sj-docx-1-tia-10.1177_23312165261436198.docx]

# Supplementary Material

to:

Measuring Spectrotemporal Sensitivity in Cochlear Implant Users with a Reaction-Time Paradigm: a Comparison of Two Implementations

Noordanus, Elisabeth^*,1^; Mens, Lucas H.M.^2^; Chalupper, Josef^3^; Balkenhol, Tobias^4^; Van Wanrooij, Marc M.^1^; Van Opstal, A. John^1^

^1^Section Neurophysics, Donders Institute for Brain, Cognition and Behaviour, Radboud University, Heyendaalseweg 135, 6525 EZ, Nijmegen, The Netherlands

^2^Department Otorhinolaryngology, Radboud University Medical Center, Geert Grooteplein Zuid 10, 6525 GA Nijmegen, The Netherlands

^3^Advanced Bionics, European Research Center, Feodor-Lynen-Straße 15, 30625 Hannover, Germany

^4^Department of Otorhinolaryngology, Heilig-Geist Hospital Bensheim, Rodensteinstraße 94, 64625 Bensheim, Germany

## S1 – LATER model

The LATER model (Linear Approach to Threshold with Ergodic Rate) describes the influence of uncertainty and internal neural noise on reaction times (Noorani & Carpenter, 2016). In the visual-motor literature this model has been successfully applied as a computational framework for decision making (Carpenter & Williams, 1995). Here, we argue that this framework may also serve as a useful tool for the analysis and interpretation of auditory-evoked reaction times under different stimulus conditions (Hofman & Van Opstal, 1998), and to reliably assess the spectral-temporal processing capacities of normal (Van der Willigen et al., 2024), vocoded (Veugen et al., 2022), and hearing-impaired listeners (Veugen, 2017).

The LATER model is depicted in Figure S1A. The green filled curve shows the typical skewed distribution of reaction times when plotted on a linear time scale. Figure S1B-C shows the cumulative response probability plotted on a probit scale against promptness (1/RT) on a linear axis — a so-called reciprobit representation. Under the LATER model, which assumes a Gaussian distribution of internal evidence accumulation rates, this transformation yields a straight line. Furthermore, it is illustrated how variations in the mean (Figure S1B) or standard deviation (Figure S1C) of the evidence rate within the model affect the outcomes. The mean reflects the perceptual difficulty of the stimulus, with a higher mean evidence rate indicating lower difficulty, whereas the standard deviation represents the level of internal noise.

| 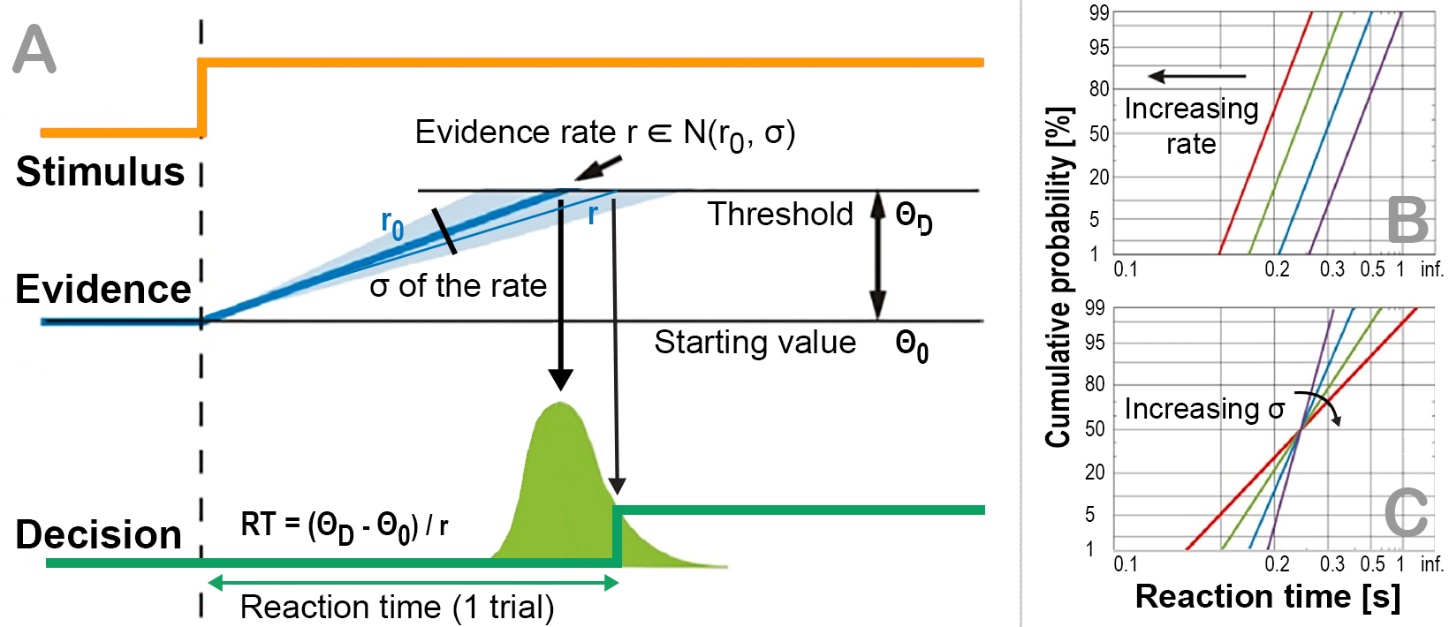  Figure S1: The LATER model. (A) In response to the stimulus, a neural decision signal rises from starting level Θ_0_ (reflecting the listener’s prior) at a constant rate, r, until the decision threshold, Θ_D_, is reached. The rate of accumulating evidence varies between trials according to a Gaussian distribution, N(r_0_, σ), with r_0_ the mean rate (thick blue line) and σ a measure for the internal noise. This causes a skewed distribution of sensory-motor reaction times (green histogram). The mean rate r_0_ corresponds to the peak of the distribution. An example trial is indicated by the thin blue line, leading to the response for this trial shown by the thick green line. (B, C) Reciprobit plots for different values of the mean (B) and standard deviation (C) of the evidence rate, respectively. Cumulative response probability (probit scale) is plotted against promptness (1/RT; x-axis labeled in reaction-time units for interpretability). After Noorani & Carpenter, 2016. |
| --- |

In the LATER model (Figure S1), the difficulty in sensory processing is reflected by the evidence rate, r, and the internal noise, σ. This internal noise likely reflects fundamental properties of neural processing circuits, which may differ between participants and may also vary across stimulus types — for example, between purely spectral, purely temporal, and combined spectrotemporal ripples. Reciprobit lines with long median reaction times indicate a low evidence rate for the associated ripples, suggesting that these stimulus modulations were more difficult to perceive (Supplementary Material, Figure S1B).

The LATER model further includes two additional parameters: the starting value of the rate, Θ_0_, and the decision threshold, Θ_D_. In fact, the reaction time in the LATER model is given by RT = (Θ_D_ - Θ_0_)/r. The starting value reflects the listener’s “prior” settings, shaped by prior knowledge — for example, knowing or expecting when and where the stimulus will appear. The decision threshold, on the other hand, is influenced by individual risk-taking or risk-avoiding strategies, as well as task-related constraints such as the emphasis on accuracy versus speed.

## S2 - Matrix test implementation

The background noise was kept constant at 60 dB SPL and the speech level was adapted, aiming for the 50% threshold of speech intelligibility in noise: the lower the outcome, the better the performance. The first sentence was presented with a signal to noise ratio (SNR) of 10 dB. The level was reduced when at least three words were repeated correctly and otherwise kept constant or increased. We used an adaptive procedure based on a maximum likelihood estimator to calculate the step sizes. The SNR change applied at the speech level for a next sentence was computed by Eqn. (1), with FC the fraction of correct answers (0, 0.2, 0.4, 0.6, 0.8 or 1), NR the number of reversals of increasing versus decreasing the SNR and T the target fraction correct. For the App implementation T= 0.6 and for the Laptop T= 0.55, the 50% speech intelligibility in noise threshold corrected for the guessing probability.

$$\Delta SNR = 10*\left( T- FC \right)\cdot\max\left( 1.5\cdot{1.41}^{NR}, 0.25 \right) (1)$$

The binominal function in Eqn. (2) gives the fraction correct for each SNR value (x in dB). After 20 sentences, this function was fitted to the measured fraction correct versus the presented SNR level.

$$FC=a_{0}+ \eta\cdot\frac{1}{1+e^{-4S\left( x-SRT \right)}} (2)$$

The free parameters for fitting Eqn. 2 are “SRT”, the 50% speech intelligibility level, and “S”, the slope of the function. The offset, *a_0_*, and gain, η, are settings of the binominal function to correct for the guessing probability of 20% for the App implementation and 10% for the Laptop implementation. The SNR level corresponding to T, the target fraction correct, gives the 50% speech intelligibility level. The table below lists the parameters.

|  | **App** | **Laptop** |
| --- | --- | --- |
| ***a_0_*** | 0.2 | 0.1 |
| **η** | 0.8 | 0.9 |
| **T** | 0.6 | 0.55 |

The best parameter combination of SRT and S is found using a differential evolution algorithm to minimize the squared distances, iterating for all possible parameters for the SRT and the slope. The optimization is based on 50 random parameter sets and stops when after 25 iterations no improvement is achieved.

The figure below shows an example of a measurement (top row) and the associated fitted binominal curve. The slope gives an indication of the reliability of the fit. For shallow slopes, below 8%/dB, the 50% speech intelligibility level is determined by averaging over the last 8 SNRs at which the speech was presented. This happened in 3% of all measurements.


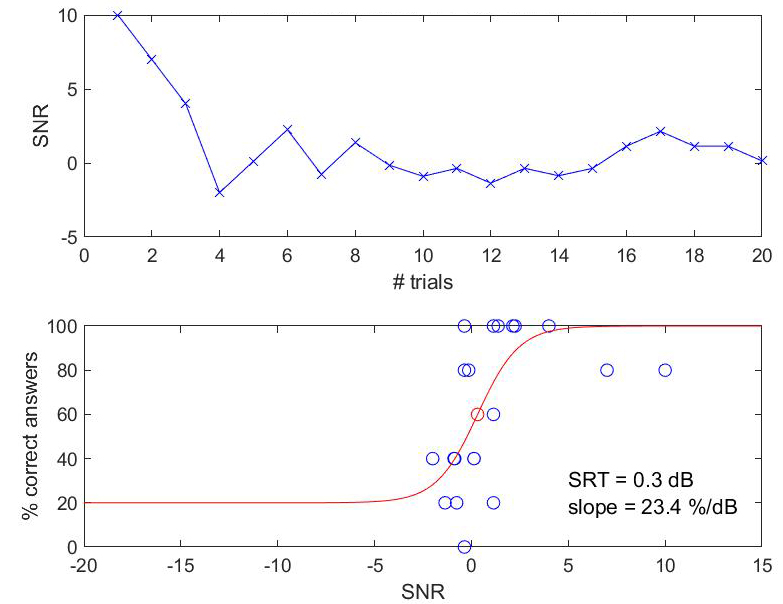


*Illustration of the determination of the 50% speech-in-noise reception thresholds for the App implementation of the Matrix test. Top row: SNR value for the 20 presented sentences. The level was reduced when at least three of the five words were repeated correctly and otherwise kept constant or increased according Eqn (1). Bottom row: fit of the percentage correct as function of the SNR value, see Eqn (2).*

## S3 – The relative delay of the App implementation

As explained in Methods, it was not feasible to directly assess the signal streams in the smartphone and Laptop to determine the absolute technical processing times. We therefore decided to determine the *relative* delay between the two implementations. To estimate the differences in delays between the Laptop and App implementations we used the psychophysical response data from all listeners (excl. s7 and s19, see Methods). The App vs. Laptop delay was then determined by a pairwise comparison per participant using Estimation Statistics of the median measured total response times for App and Laptop. This analysis was performed on the median (per participant and stimulus) reaction times for three easily perceived stimuli - [4 Hz, 0.5 c/o], [8 Hz, 0 c/o], [16 Hz, 0.25 c/o] - to which all participants responded with a low number of outliers for either implementation (Figure 4A). The results are shown in the below figure. The mean delay for the App measurement was 450 ms, credible interval: [426 – 475 ms]. We subtracted this delay from the App responses and applied the processing (removing responses faster than 140 ms and setting RTs exceeding 2500 ms and non-responses to 2500 ms, see Methods). To refine the calculation of the delay, we then made Gaussian fits of the resulting distributions of the promptness (1/RT) for the three easily perceived stimuli. We used the promptness instead of the reaction times because the promptness typically follows a Gaussian distribution (LATER model, see Introduction). The mean of the App distributions was (transformed to reaction times) 20 ms higher than that of the Laptop. We thus obtained a definitive delay in the reaction times for the App measurements of 470 ms.


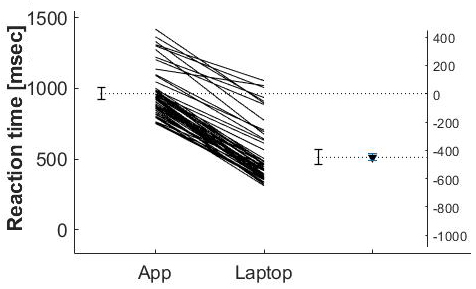


*Paired mean difference plot of the median reaction times for App vs. Laptop, for the three easy spectrotemporal ripples for each participant (N=57 responses for each implementation). The mean across participants shows that the Apps’ recorded response times were delayed by 450 ms (CI: [426-475] ms) with respect to the Laptop.*

To estimate the Bluetooth latency, we compared response-time measurements for a normal hearing participant using a Bluetooth headphone (Sony WH-H900N) and the built-in iPhone 6s speaker. This delay was found to be 210 ms. The additional delay introduced by the ComPilot is around 60 ms. The remaining portion of the 470 ms delay is likely attributable primarily to the iPhone-6s touchscreen-sensing delay, as well as to minor differences in overall response strategy (e.g., precise vs. fast), response speed when tapping a touchscreen versus pressing a spacebar, and acoustic factors (see Discussion).

## S4 - Influence of implementation, stimulus type, and participant

In addition to the main analysis — based on median reaction times with credible intervals derived using Estimation Statistics (Analysis method 1, Methods) — we also performed a Bayesian analysis (Analysis method 2) to better disentangle the contributions of implementation, stimulus, and participant. This complementary analysis is well suited to datasets with substantial numbers of outliers and censored observations (e.g., non-responses). Specifically, we fitted a Bayesian hierarchical generalized linear model (Kruschke, 2015) that included the main effects of implementation, participant, and stimulus, as well as all first-order interactions. The approach matches that used in Noordanus et al. (2025), where similarly high proportions of censored reaction-time data were analyzed. This model provides posterior estimates and 95% credible intervals for each effect, thereby quantifying their relative contributions.

The model predicts the metric promptness, described as a Gaussian distribution, using as nominal predictors: Implementation (I), Stimulus (S), and Participant (P). The factor “Implementation” had two levels (App and Laptop), the factor “stimulus” had 17 levels (Table 2) and the factor “Participant” had 19 levels (Table 1, excluding S7 and S19). The mean of the predicted promptness ($\bar{Pr}$) for a specific combination of Implementation, target electrode and participant (s, e, p) can be calculated as the sum of the means of the Stimulus type, target Electrode and Participant deflections. Here, “deflection” refers to the effect of each factor, representing the difference from the promptness across all responses:

$$\bar{Pr}_{\Delta MF}\left( i,s, p \right)={Pr}_{0}+\delta I(i)+\delta S(s)+\delta P(p) +\delta IS(i,s)+\delta SP(s,p)+\delta IS(i,s) (S4\_1)$$

with Pr_0_: mean of the predicted promptness for all trials, δI(i): Implementation deflection, δS(s): Stimulus deflection, δP(p): Participant deflection, δIS(i,s): deflection of the Implementation – Stimulus interaction for implementation i, and stimulus s, δSP(s,p): deflection of the Stimulus – Participant interaction for stimulus s, and participant p, and δIS(i,s): deflection of the Implementation – Stimulus interaction for implementation i, and stimulus s. Each of the terms on the right-hand side of Eqn. (S4_1) is the average of 50,000 sample steps in the analysis. The model as shown in Figure S4.1 in Supplementary Material S4 in Noordanus et al. (2025) was used ([download link](https://ars.els-cdn.com/content/image/1-s2.0-S0378595525001935-mmc1.docx)). The model was fitted using Monte Carlo Markov Chain sampling in MATLAB (The MathWorks, Natick, MA, USA, version R2023a), using JAGS v4.3.0 (Plummer, 2017^[[1]](#footnote-2)^), and MATJAGS, a MATLAB interface for JAGS.

The model was estimated using 50,000 samples following a burn-in period of 20,000 iterations, with four chains run in parallel. Convergence and sampling adequacy were assessed both qualitatively and quantitatively: trace plots were inspected visually, and diagnostics were evaluated by computing the lowest equivalent sample size across parameters and the highest shrink factor, following the recommendations of Kruschke (2015). As a guideline, effective sample sizes exceeding 1,000 and scale reduction factors below 1.1 indicate satisfactory convergence. These criteria were met for all model parameters.

The results of Analysis method 2 are summarized in Figure S4. Overall, the Bayesian hierarchical model fully supports the conclusions drawn from the main analysis: implementation differences are small, stimulus effects are robust, and the dominant structure in the data reflects consistent individual differences in sensitivity across stimuli.

- Panel A: **main effect of implementation**. A small effect (0.04 s^-1^) was observed, corresponding to ~40 ms (given the overall mean RT of 690 ms). Analysis method 2 estimates this using all stimuli (after correction App data for the delay, similar to Analysis method 1, see Supplementary Material S3) , whereas Analysis method 1 derived the correction from three selected reference stimuli (Supplementary Material S3).
- Panel B: **main effect of stimulus**. The stimulus-dependent pattern closely matches Figures 4A and 5 in the manuscript. Conclusions are identical to those from Estimation Statistics, with two minor differences: the [4 Hz, 0.5 c/o] – [8 Hz, 0.5 c/o] comparison reaches significance, and the [16 Hz, 0.25 c/o] stimulus is inferred to be significantly faster than the others.
- Panel C: **main effect of participant**. The participant effect spans a range comparable to the stimulus effect (panel B), consistent with the inter-participant spread visible in Figure 4A.
- Panel D: **implementation x stimulus interaction**. Differences between App and Laptop conditions are small, in line with Figures 4B–C. The interaction’s effect size is much smaller than that of the participant × stimulus interaction (panel E), indicating that implementation differences do not explain the stimulus-dependent response pattern.
- Panel E: **participant x stimulus interaction**. Substantial within-participant variation across stimuli is evident, consistent with the patterns shown in Figures 2 and 3.
- Panel F: **participant x implementation interaction**. Most participants exhibit an App–Laptop difference of ~40 ms, with S2 (App faster by 180 ms) and S20 (Laptop faster by 113 ms) showing the largest deviations.

| 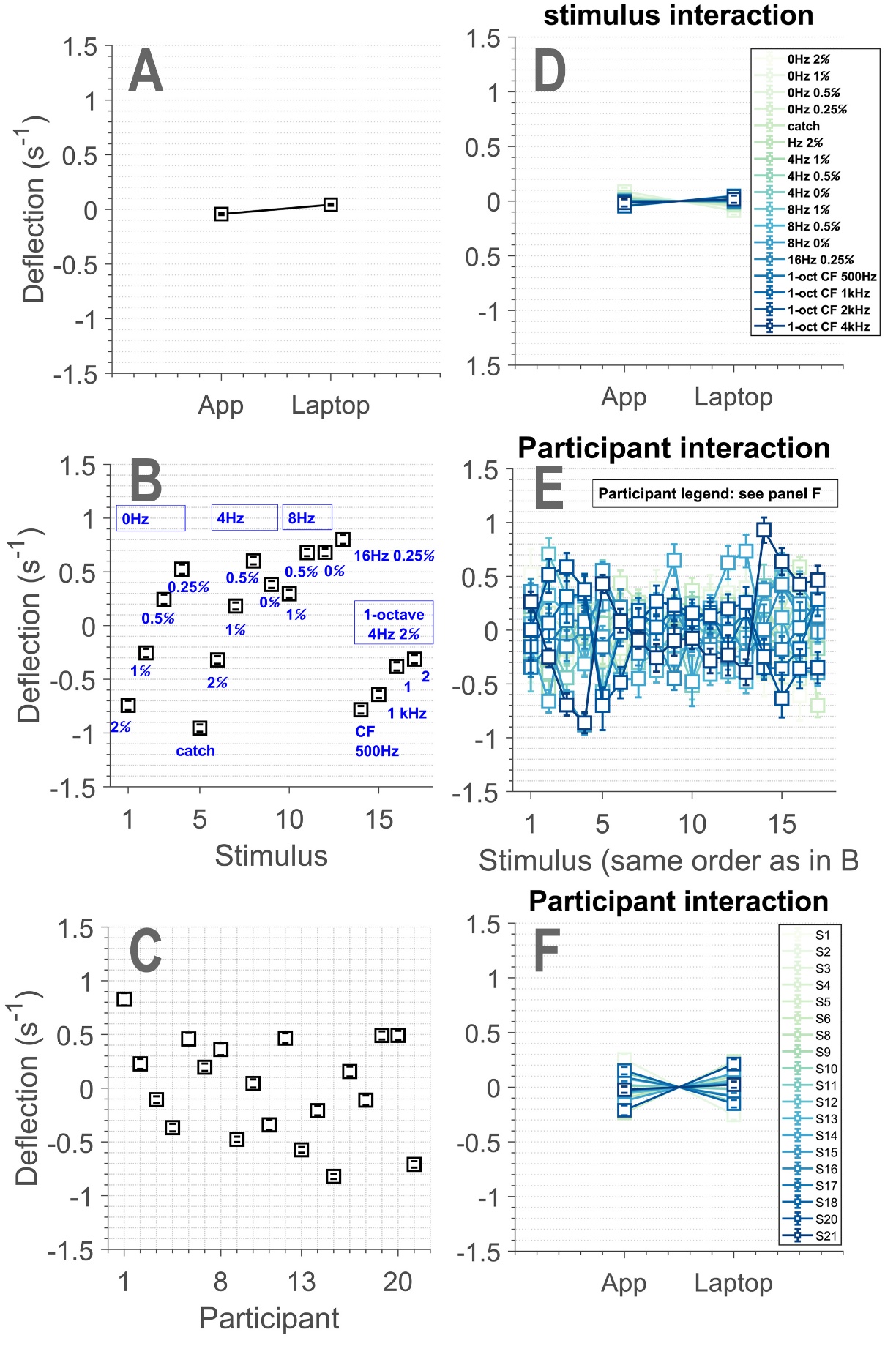 |
| --- |
| *Figure S4:* ***Bayesian hierarchical generalized linear model estimating main effects and first-order interactions****. The left column shows the main effects of implementation, stimulus, and participant; the right column shows their corresponding first-order interactions. Deflections indicate deviations from the mean promptness across all responses. Boxes denote posterior means, and error bars represent 95% credible intervals. The ordering of stimuli in panels B and D follows Figure 4, except that in Figure 4 the catch stimulus is placed at the end.* |

## S5 – Reciprobit plots for the narrowband stimuli


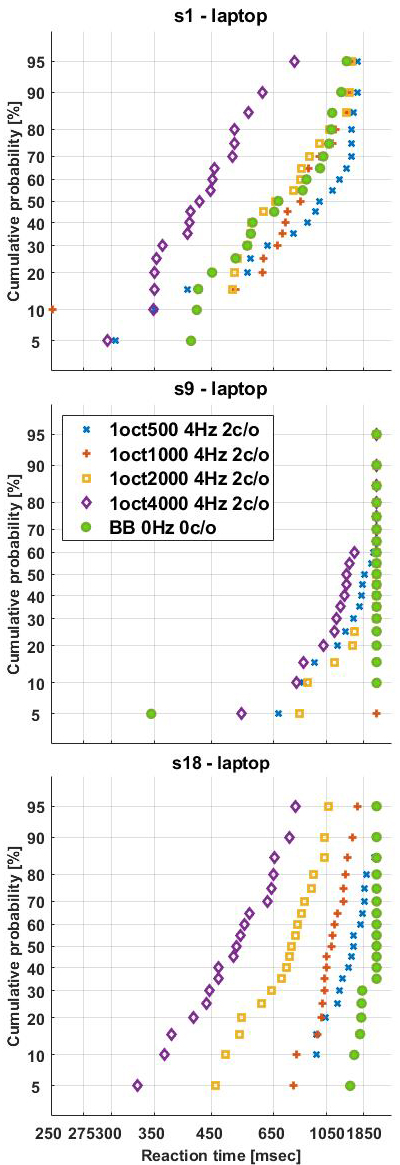


*Example reciprobit plots with narrowband stimuli (1-octave center frequency 500 Hz, 1, 2, and 4 kHz) in the Laptop implementation for three participants. In reciprobit plots (see Methods), cumulative response probability (probit scale) is plotted against promptness (1/RT), allowing stimulus-specific reaction-time distributions to be visualized and compared. Data from S9 are also provided in Figure 3.*

*The narrowband 4 kHz stimulus (purple diamonds) elicited the fastest responses for the three participants, with the narrowband 500 Hz ripple (blue crosses) yielding the longest reaction times. Participant S18 (bottom) was the best speech performer of the cohort; the reciprobit lines for this listener are clearly separated. Note that S1 (top) had slower responses for the narrowband 500 Hz stimulus than for the catch stimulus (green symbols). Participants S9 and S18 did not respond to the catch stimulus.*

## S6 – Responses to the catch trials

Figure S6 shows the median reaction times for the catch stimulus for all participants, comparing the App and Laptop implementations. Three participants exhibited late false-positive responses, with median reaction times between 1500 and 2500 ms for both implementations. Two participants (S1 and S5) showed markedly shorter median reaction times of approximately 700 ms for the catch stimulus, again for both methods. These responses were still slower than those elicited by all other stimuli except the narrowband 500 Hz ripple (see Supplementary Material S5). The median difference in false-positive reaction times between the App and Laptop was zero. Importantly, for 19 of the 21 participants, the catch stimulus elicited the slowest reaction time of all conditions.

Two participants responded to none or only one of the 40 catch trials (20 per implementation). Excluding the five participants noted above, the true-negative rate for the catch stimulus was 76%.

| 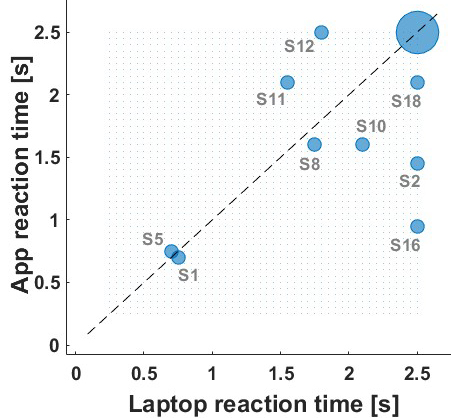 |
| --- |
| *Figure S6: Bubble plot of the median reaction time to the catch stimulus [0 Hz, 0 c/o] for all participants (excl. S7 and S19) for Laptop versus App. The grid resolution is indicated by the tiny dots.* |

## S7 - Spectrotemporal MTF for Laptop and App


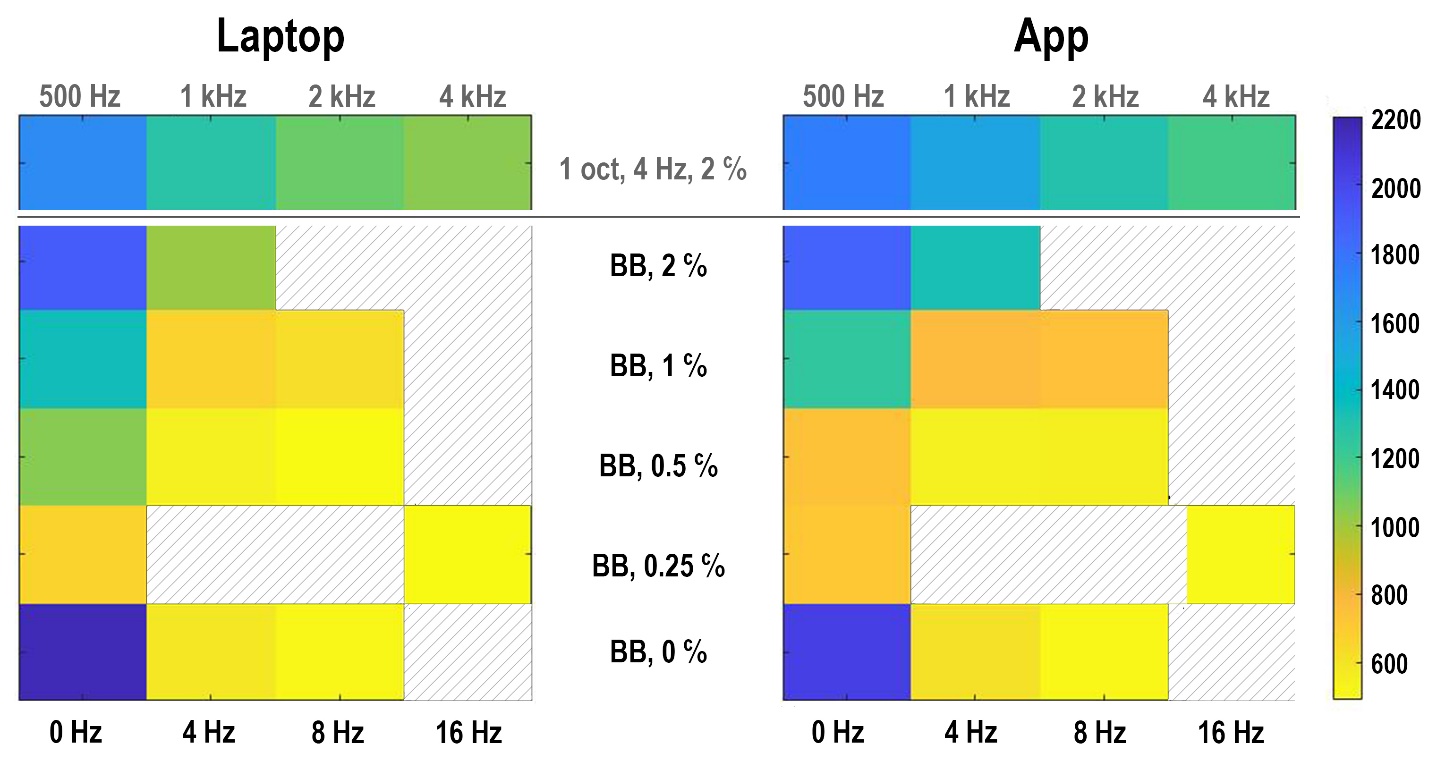


*Average* *of the median reaction times per stimulus across participants, shown separately for the Laptop (left) and App (right) implementations. The top color box row shows the averages for the narrowband [4 Hz, 2 c/o] stimuli, the center frequency is indicated above in grey font. The other rows show the averages for the broadband stimuli (stimulus order is the same as in Table 2). The averages of the medians across participants for each stimulus are very similar for the Laptop and App implementation. Only the [0 Hz, 0.5 c/o] is clearly different between Laptop and App, this stimulus is also significantly different when taking all RTs in account (Figure 4B). Note that these matrix plots summarize the joint spectral-temporal sensitivity of the listeners. The modulation transfer functions shown in Figure 5 correspond directly to the rows (temporal MTF) and columns (spectral MTFs) of these plots. However, whereas Figure 5 depicts the mean and standard deviation based on all individual data points, the plots here are based on participant-wise medians.*

## S8 – App speech-in-noise results and App - STM-RT correlation

### Speech-in-noise results with the App

An adapted version of the Matrix speech-in-noise test was incorporated in the TRaM app. As mentioned in the Methods, all App measurements were performed with the same iPhone 6s mobile phone. The App speech-in-noise test was performed in the same session as the App STM-RT test, that is, two to three months earlier than the Laptop speech-in-noise test (Methods).

The speech-in-noise matrix test as described in the Methods served as the basis for the App implementation. For the App implementation, audio was streamed via Bluetooth to a Phonak ComPilot. The ComPilot then transmitted the signals to the CI sound processor via 10.6 MHz radio frequency technology.

To adapt the app for smaller screen sizes, the original 10×5 word matrix used in the laptop version (printed on an A4 sheet) was replaced with a more compact 5×5 matrix. Notably, each test presented a unique arrangement of words, with the correct word for each column randomly placed in one of the rows. The remaining words in each column were randomly selected from the other nine options. Figure S8_1 provides a screenshot illustrating an example of the app's implementation. Participants selected the perceived words by tapping one word in each column. If no word was selected, the app automatically chose a random word. Consequently, the guessing probability was 1/5 for the app. The calculation of the 50% speech reception threshold (SRT) was adjusted to account for a higher guessing probability of 20% (app) versus 10% (laptop), see Supplementary material S2.

Similar to the laptop implementation (Methods), noise level was fixed at 60 dB SPL, while the speech level was adapted to target the 50% SRT. The first sentence was presented with a signal-to-noise ratio (SNR) of +10 dB, and the speech level was reduced if at least three words were correctly identified.

| 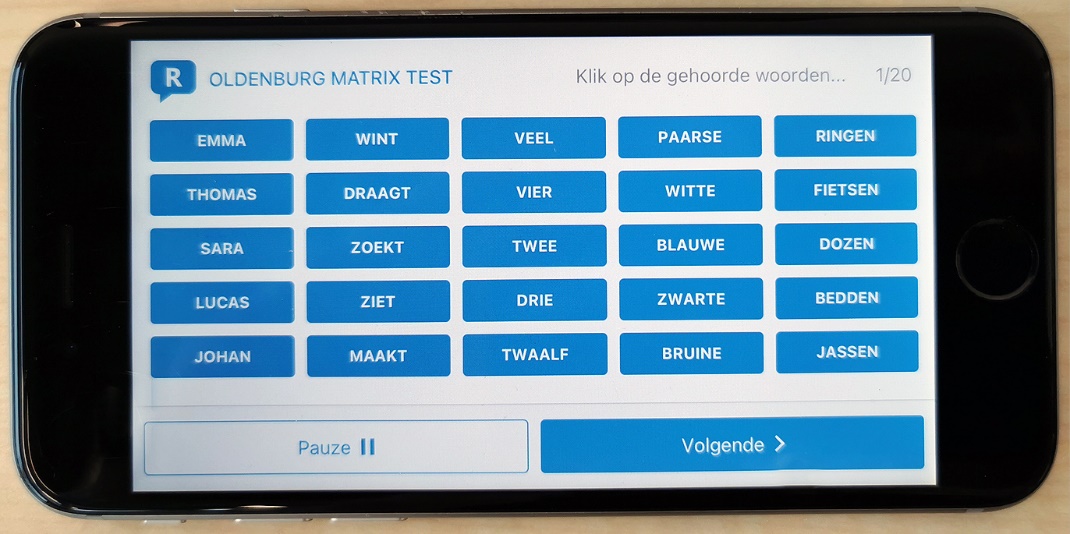 | *Figure S8_1: Matrix speech-in-noise implementation in the TRaM app (see Methods).* |
| --- | --- |

| 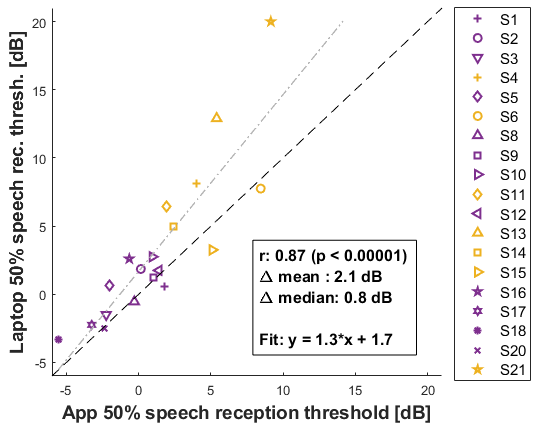 | Figure S8_2: Speech-in-noise thresholds obtained in the lab via the App- and Laptop-based methods, with each data point representing the mean of two App and two Laptop tests for a single participant. The dashed line represents the x=y reference, while the light grey dash-dotted line shows the fitted regression line. |
| --- | --- |

Figure S8_2 compares the speech-in-noise results obtained in the laboratory using the app with those obtained using the Laptop-based standard implementation (Figure 6). Each data point represents the mean of two tests for each method. Test-retest repeatability was 1.2 dB for the app and 0.9 dB for the laptop. A strong Pearson correlation was observed between the app and laptop measurements (r=0.87), explaining 75% of the variance. On average, participants performed better using the app compared to the laptop. Notably, the laptop results showed a trend of declining performance at higher presentation levels. When excluding the five participants whose 50% speech reception thresholds exceeded 5 dB in the laptop measurement, this trend disappeared (fit: y = 0.7x + 0.9), while the correlation remained similar (r = 0.83, p = 0.0003).

The worse performance at higher presentation levels may be attributed to the free-field presentation of stimuli in the standard version, as opposed to the direct audio streaming to the sound processor in the app-based implementation. The benefit of direct audio streaming is supported by Schafer and Kleineck (2009). Additionally, the reduced number of answer options in the app-based test (5x5 vs. 10x5, see Methods) may have further contributed to the observed performance differences.

### Correlation between STM-RT and speech in noise

Spearman’s rank correlations were calculated between speech-in-noise thresholds and STM-RTs for each of the ripple types. The results are shown in Figure S8_3 for the App (left) and Laptop (center). For transparency, uncorrected p-values are shown in this supplementary figure (*: *p* < 0.05: **: *p* < 0.01), whereas the main text (Figure 8) displays only correlations meeting the more conservative criteria (95% credible interval excluding zero), with three asterisks (***) indicating survival of Holm–Bonferroni correction. The same Holm–Bonferroni criterion (***) is applied in this supplementary figure. In general, correlations between STM-RTs and speech-in-noise perception were highly similar for the App and Laptop methods, even though the measurements were performed in different sessions two to three months apart.

The Laptop results shown here are based on identical data as the Laptop results in Figure 8 in the main text. Figure 8 shows only asterisks for correlations whose 95% credible interval excluded zero, this was the case for all stimuli with p < 0.05, p not corrected for multiple comparisons, except two ([0 Hz, 0.5 c/o] and the narrowband stimulus with center frequency 2 kHz). The correlation pattern for the App obtained with Laptop speech-in-noise results (Figure 8) is identical to that obtained with App speech-in-noise results (Figure S8_3, left panel). Correlation values obtained with the Laptop speech-in-noise results are in general somewhat higher than those obtained with App speech-in-noise results.

| 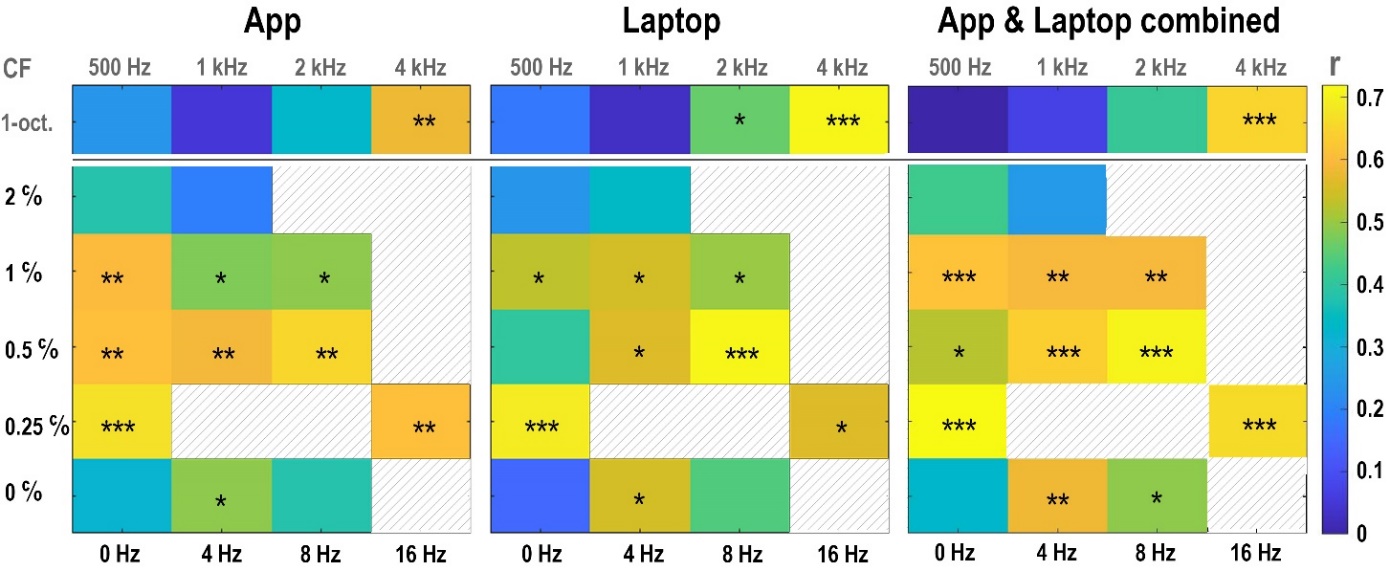  Figure S8_3: Spearman’s rank correlation, r, between STM-RT and speech-in-noise perception, analyzed for App data only (left panel), Laptop data only (middle panel), and for the combined data (right panel). Participants S7 and S19 are excluded from the App only and the Laptop only analysis; in the combined App & Laptop analysis all subjects are included. The top row of colored boxes shows the correlations for the 1-octave [4 Hz, 2 c/o] stimuli (the center frequency CF is indicated above). The other five rows show the correlations for the broadband stimuli (stimulus order is the same as in Table 2). Maximum of the color bar: r = 0.72. *: p<0.05, **: p<0.01, p not corrected for multiple comparisons. ***: significant after performing Holm-Bonferroni correction for testing 17 stimuli. Note that the middle panel is identical to the right panel of Figure 8 in the main text, except that there one asterisk denotes 95% credible interval excluding zero. |
| --- |

To obtain more stable participant-specific estimates of reaction times and speech-in-noise thresholds, we additionally averaged the App and Laptop measurements within participants. We included the available measurements for participants S7 and S19 but excluded the App STM-RT measurements for S19 (see caption Figure 2). The right-hand panel in Figure S8_3 shows that the correlations between the STM-RT and speech-in-noise results were slightly stronger when using the combined Laptop and App results, with correspondingly more ripple types surviving Holm–Bonferroni correction.

Of the broadband stimuli, only the 2 c/o stimuli had no significant correlation with speech in noise. As described in the Introduction, this finding is in line with Elliot and Teunissen (2009), who reported that speech is best represented by spectral modulations < 1.0 c/o and ripple velocities below 10 Hz. Of the 1-octave [4 Hz, 2 c/o] stimuli, only the ripple with center frequency 4 kHz correlated significantly with the speech-in-noise reception thresholds.

Strongest correlations were obtained for six ripples (***), each significant after performing Holm-Bonferroni correction for testing 17 stimuli, with coefficients of determination in the range r^2^ = 0.37 - 0.53.

## S9 – Design factors reaction time implementation

In addition to the factors mentioned in the paragraph “Measuring reaction times” in the Discussion, the following experimental design factors are important to consider for an adequate assessment of stimulus-evoked reaction times.

### **Unpredictability**

- The ripple onset must be adequately randomized from trial to trial to avoid prediction of the upcoming stimulus event. For example, the variability in the pre-ripple static-noise duration should be large enough (suggested range: 1.5–3.5 seconds) to prevent build-up of an internal rhythm that would increase the prior level, Θ_0_.
- Different ripples should be randomly interleaved to prevent adaptation effects and prediction.
- A *catch stimulus* without modulation [0 Hz, 0 c/o] must be presented in about 5% of the trials, randomly interleaved with the test stimuli. Responses to this stimulus (‘false positives’) give an estimation of the overall response readiness and guess rate of the participant. A reciprobit plot (Methods) is very useful for identifying false positives because true responses show up as a straight line.

### **Training**

- The task instruction should be clear and unambiguous and should emphasize the importance of speed over precision. To keep motivation levels high, participants could receive some form of positive feedback about their reaction time after each trial, and/or see a progress bar indicating their progress in the session.
- Before starting, a training session is needed to familiarize the listener with the response procedures and the stimuli. Otherwise ‘procedural learning’ effects may show up (De Jong et al., 2018), resulting in a gradual decrease of overall reaction times. Typically, stable within-day performance can be achieved after a few tens of trials, but sometimes listeners (especially poor speech-in-noise performers) may require longer to yield stable behavior. In the LATER model, procedural learning is represented as a lowering of the decision threshold Θ_D_.

### Stimulus quality

- The participant should be able to perceive the full range of the modulations, including the troughs, which must fall within the CI user's dynamic range. If the dynamic range is insufficient, the stimulus level and modulation depth should be adjusted accordingly.
- Care should be taken to avoid false acoustic cues during stimulus presentation, which could trigger a response from the participant that is not evoked by the relevant acoustic (spectral-temporal) modulation. For example, the onset of AGC induces a sudden decrease of sound level. By having its onset sufficiently long before ripple onset (e.g., >1.5 seconds), participants can readily learn to avoid responding to the AGC during the brief training session. Alternatively, the level should ideally be set such that the automatic gain control (AGC) is not activated.
- The catch stimulus also serves to identify potential artifacts in the stimulus-generation and those introduced in the CI processor.

### **Accuracy**

- Reaction times should be measured with sufficient temporal resolution. Given the subtle differences in reaction times to different ripples (in the order of a few tens of ms), the precision should be such that the error in the determination of the median reaction time is within ± 10 ms. Note that the response mode (e.g., small versus large button) also contributes to the variability of the response time.
- To ensure that the reaction time is due to neural processing and that measurements from different setups and laboratories can be quantitatively compared, any additional delay, such as the Bluetooth delay, electronic delays in the response measure (e.g., spacebar/laptop vs. touch screen/smartphone), etc., should be corrected for.

## S10 – Electrodograms

We simulated CI electrode stimulation patterns using the Generic MATLAB Toolbox (GMT; <https://github.com/jabeim/GMT>), as recommended by Advanced Bionics. GMT is an open-source collection of MATLAB scripts that reproduces the signal-processing stages of Advanced Bionics cochlear implant systems.

Panel A-C in Figure S10_1 show electrodograms of the broadband stimuli of which Figure 1A-C shows the spectrograms: [4 Hz, 0 c/o], [0 Hz, 2 c/o] and [4 Hz, 0.5 c/o], respectively. Panel D shows the broadband [4 Hz, 2 c/o]. A selection of the other broadband stimuli is shown in Figure S10_3.

| 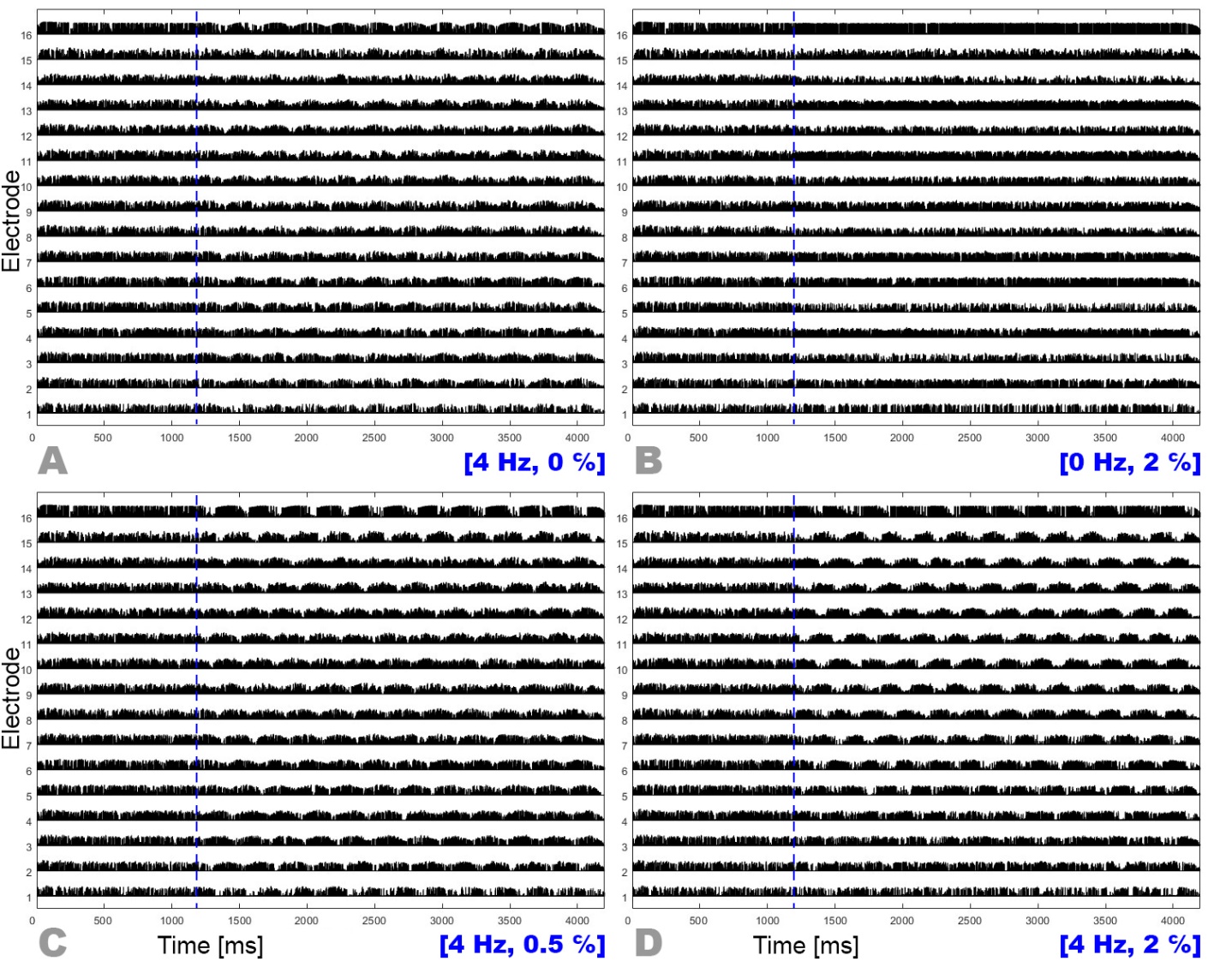 |
| --- |
| *Figure S10_1: Electrodograms generated using the Generic MATLAB Toolbox (GMT) for the broadband stimuli. Vertical dashed lines at 1200 ms indicate modulation onset. Panels A–C correspond to the same stimulus conditions shown in the spectrograms of Figure 1A–C: (A) [4 Hz, 0 c/o], (B) [0 Hz, 2 c/o], and (C) [4 Hz, 0.5 c/o]. Panel D shows the broadband [4 Hz, 2 c/o] stimulus. The modulation patterns are clearly represented across electrodes, with less distinct pulse-pattern fluctuations in the lower-frequency channels.* |

Figure S10_2 presents the electrodograms for the narrowband (1-octave) versions of the [4 Hz, 2 c/o] stimulus (see panel D in Figure S10_1), with center frequencies of 4 kHz, 2 kHz, 1 kHz, and 500 Hz for panels A–D, respectively. The 15 dB accentuation of the ripple band is visible in each panel. Pulse patterns are markedly more distinct in the higher-frequency channels (panel A and B) than in the low-frequency channels (panel D). For the 1 kHz stimulus (panel C), the modulation is already less clearly represented — particularly around electrode 5 — illustrating the reduced salience of modulation cues in lower-frequency channels. See also the discussion of narrower low-frequency filters in the “Modulation transfer functions” section of the Discussion.

| 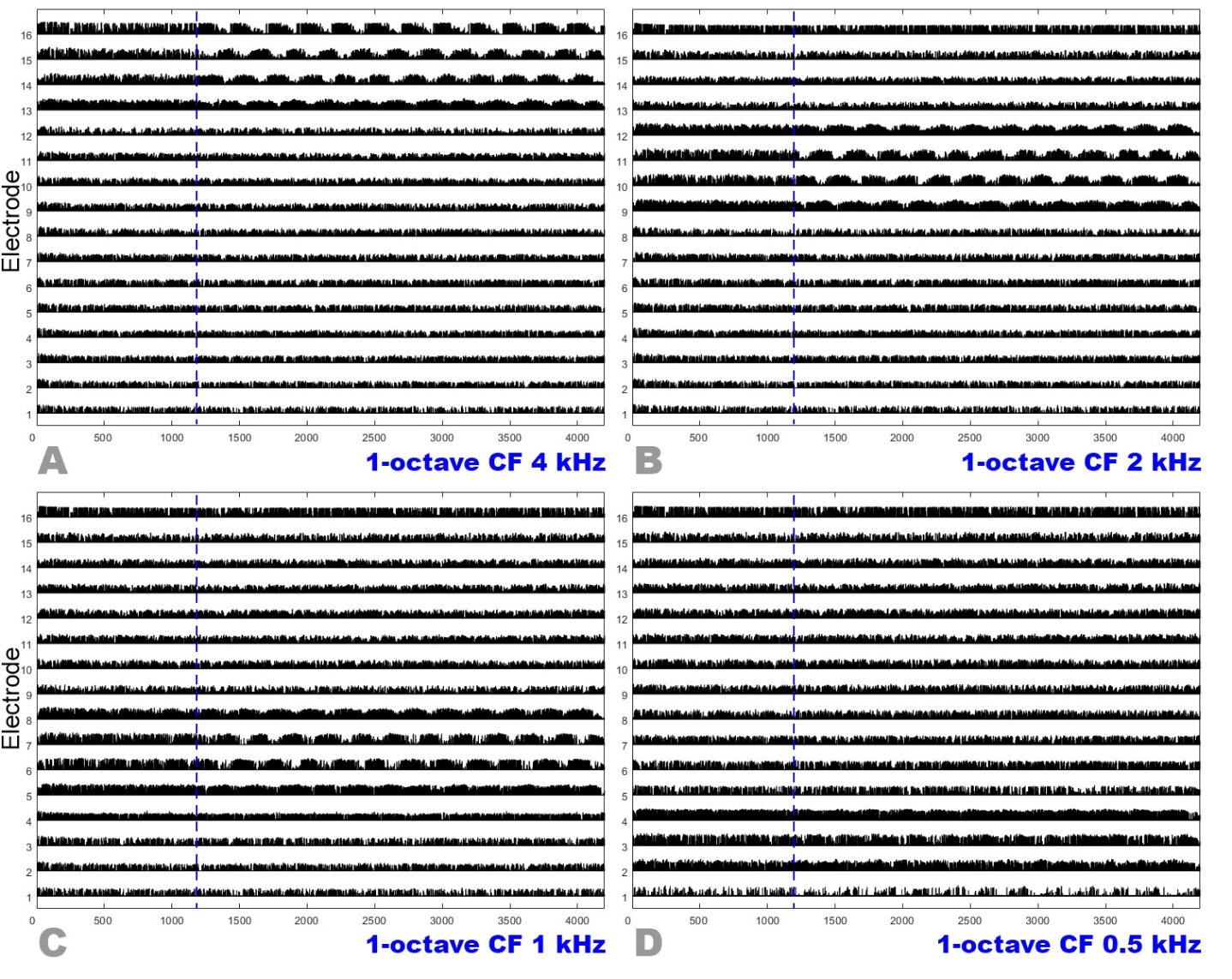 |
| --- |
| *Figure S10_2: Electrodograms for the narrowband (1-octave) versions of the [4 Hz, 2 c/o] stimulus, simulated using the Generic MATLAB Toolbox (GMT). Vertical lines indicate modulation onset. Panels A–D show stimuli centered at 4 kHz, 2 kHz, 1 kHz, and 500 Hz, respectively. The 15 dB accentuation of the ripple band is visible in each case. Modulation cues are markedly more distinct in the high-frequency channels (panel A) than in the low-frequency channels (panel D). At 1 kHz (panel C), the modulation becomes less clearly represented — especially at electrode 5 — illustrating how lower-frequency channels yield less salient modulation patterns..* |

Figure S10_3 shows electrodograms for a selection of the broadband stimuli not included in Figure S10_1. Panel A displays the purely temporal 8Hz, which elicited the fastest responses in most participants with the highest speech-in-noise thresholds (see Discussion). Panel C shows the corresponding spectrotemporal stimulus, [8 Hz, 0.5 c/o], which yielded a similar average reaction time to the purely temporal 8 Hz condition (Supplementary Material S4, Figure S4B), but exhibited greater between-participant variability (Figure 4A).

All 12 good and medium speech-in-noise performers had their fastest responses to the [0 Hz, 0.25 c/o] or the [16 Hz, 0.25 c/o] ripple (see Discussion), shown in panel B and D, respectively.

| 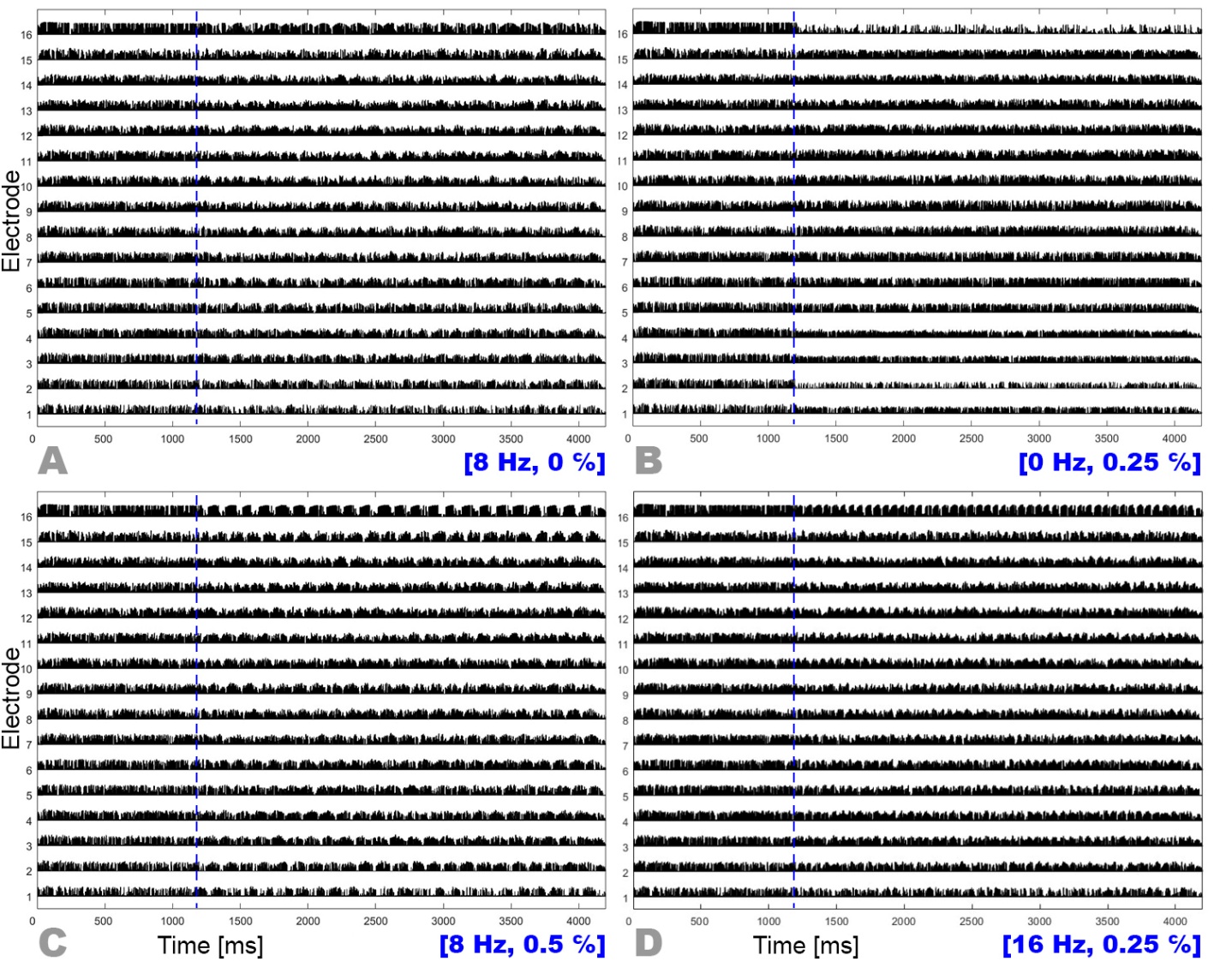 |
| --- |
| *Figure S10_3: Electrodograms generated using the Generic MATLAB Toolbox (GMT) for the broadband 8Hz stimuli. Vertical lines indicate modulation onset. Further see text.* |

1. Plummer, M., 2017. JAGS Version 4.3.0 user manual. Retrieved 2024, from https://people.stat.sc.edu/hansont/stat740/jags_user_manual.pdf. [↑](#footnote-ref-2)
